# The relationship between hair metabolites, air pollution exposure and gestational diabetes mellitus: A longitudinal study from pre-conception to third trimester

**DOI:** 10.3389/fendo.2022.1060309

**Published:** 2022-12-02

**Authors:** Xuyang Chen, Xue Zhao, Mary Beatrix Jones, Alexander Harper, Jamie V. de Seymour, Yang Yang, Yinyin Xia, Ting Zhang, Hongbo Qi, John Gulliver, Richard D. Cannon, Richard Saffery, Hua Zhang, Ting-Li Han, Philip N. Baker

**Affiliations:** ^1^ Department of Obstetrics and Gynaecology, The First Affiliated Hospital of Chongqing Medical University, Chongqing, China; ^2^ Chongqing Key Laboratory of Maternal and Fetal Medicine, Chongqing Medical University, Chongqing, China; ^3^ Department of Statistics, The University of Auckland, Auckland, New Zealand; ^4^ Department of Health Sciences, University of Leicester, Leicester, United Kingdom; ^5^ College of Health, Massey University, Auckland, New Zealand; ^6^ School of Public Health and Management, Chongqing Medical University, Chongqing, China; ^7^ Chongqing Key Laboratory of Oral Diseases and Biomedical Sciences, Stomatological Hospital of Chongqing Medical University, Chongqing, China; ^8^ Department of Obstetrics and Gynecology, Women and Children’s Hospital of Chongqing Medical University, Chongqing, China; ^9^ Centre for Environmental Health and Sustainability & School of Geography, Geology and the Environment, University of Leicester, Leicester, United Kingdom; ^10^ Department of Oral Sciences, Sir John Walsh Research Institute, Faculty of Dentistry, University of Otago, Dunedin, New Zealand; ^11^ Molecular Immunity, Murdoch Children’s Research Institute, Royal Children’s Hospital, Melbourne, VIC, Australia; ^12^ Liggins Institute, The University of Auckland, Auckland, New Zealand; ^13^ Department of Obstetrics and Gynaecology, The Second Affiliated Hospital of Chongqing Medical University, Chongqing, China; ^14^ College of Life Sciences, University of Leicester, Leicester, United Kingdom

**Keywords:** gestational diabetes mellitus, cohort study, metabolomics, hair analysis, air pollution

## Abstract

**Background:**

Gestational diabetes mellitus (GDM) is a metabolic condition defined as glucose intolerance with first presentation during pregnancy. Many studies suggest that environmental exposures, including air pollution, contribute to the pathogenesis of GDM. Although hair metabolite profiles have been shown to reflect pollution exposure, few studies have examined the link between environmental exposures, the maternal hair metabolome and GDM. The aim of this study was to investigate the longitudinal relationship (from pre-conception through to the third trimester) between air pollution exposure, the hair metabolome and GDM in a Chinese cohort.

**Methods:**

A total of 1020 women enrolled in the Complex Lipids in Mothers and Babies (CLIMB) birth cohort were included in our study. Metabolites from maternal hair segments collected pre-conception, and in the first, second, and third trimesters were analysed using gas chromatography-mass spectrometry (GC-MS). Maternal exposure to air pollution was estimated by two methods, namely proximal and land use regression (LUR) models, using air quality data from the air quality monitoring station nearest to the participant’s home. Logistic regression and mixed models were applied to investigate associations between the air pollution exposure data and the GDM associated metabolites.

**Results:**

Of the 276 hair metabolites identified, the concentrations of fourteen were significantly different between GDM cases and non-GDM controls, including some amino acids and their derivatives, fatty acids, organic acids, and exogenous compounds. Three of the metabolites found in significantly lower concentrations in the hair of women with GDM (2-hydroxybutyric acid, citramalic acid, and myristic acid) were also negatively associated with daily average concentrations of PM_2.5_, PM_10_, SO_2_, NO_2_, CO and the exposure estimates of PM_2.5_ and NO_2,_ and positively associated with O_3_.

**Conclusions:**

This study demonstrated that the maternal hair metabolome reflects the longitudinal metabolic changes that occur in response to environmental exposures and the development of GDM.

## Introduction

Industrialization accelerated the consumption of coal combustion and fossil fuels for transportation and other human activities. Environmental pollution, in particular air pollution, has become a major public health issue and a leading cause of disease especially in developing countries (1, 2). In China, air pollution was estimated to cause approximately 1.24 million deaths and a reduction in the mean life expectancy by about 1.25 years in 2017 (3). Air pollution is also associated with numerous inflammatory diseases that frequently arise with comorbidities, such as chronic obstructive pulmonary disease, cancer, metabolic diseases, and diabetes (4–8).

Pregnancy and early infancy are considered to be highly sensitive periods where exposures could lead to lifelong consequences (9). Pregnant women are exposed to hundreds of chemicals at low levels and these exposures could operate additively or interactively, raising the possibility of ‘mixture’ effects (10). Exposure to air pollution in pregnancy has been linked to a range of adverse outcomes, including miscarriage, pre-eclampsia, preterm birth, birth to an infant small for gestational age, and gestational diabetes mellitus (GDM) (11–17). Pregnancy involves a dynamic flow of metabolic changes week by week, with substantial physiological alterations in glucose and lipid metabolism (18). Pregnant women are particularly prone to abnormal glycaemia as elevated insulin resistance is part of the normal physiological adaptation to pregnancy, to ensure greater substrate availability for fetal growth (19). However, up to 20% of all pregnancies develop some degree of impaired glucose tolerance in China (20). GDM is associated with serious short-term and long-term adverse health consequences for both the mother and baby (21–23). Although the precise pathogenesis of GDM remains unclear, GDM shares similar pathophysiological features with type 2 diabetes mellitus (24). Several studies have reported that air pollution is an important risk factor for type 2 diabetes mellitus, through inflammation-related insulin resistance, endothelial dysfunction, and dysregulation of adipose tissue (8, 25–27). Genome-wide association studies indicate that genetic factors contribute to only a small proportion of GDM risk, and the increased prevalence of GDM has occurred with minor to no shift in the genetic composition of the population (28, 29). Specific air pollution exposures potentially associated with increasing GDM risk include exposure to particulate matter less than 10 μm in diameter (PM_10_), NO_2_, and SO_2_, among others (30–34). The timing of air pollution exposure in pregnancy may impact GDM risk but data are scarce and often contradictory (35). Therefore, it is of great significance to study the biological effects of environmental exposures during pregnancy and early infancy.

The burden of the risk attributable to environmental factors is unclear, and this is in part due to the limited capacity to accurately measure the complex mix of pollutants and other environmental exposures across an extended timeframe. Metabolomics is a powerful approach for directly identifying and quantifying low molecular weight compounds. Metabolomics aims to view the complex nature of how physiology is related to external exposure, and how these associations may be linked to disease outcomes (36–38). Although epidemiological studies have shown associations between air pollution and increased GDM risk, few studies have linked these associations to longitudinal metabolic changes *in vivo*. Conventional samples used in metabolomics studies, such as urine and blood, can be influenced by acute and transient factors such as recent dietary intake and immune status (39, 40). The dynamic nature of conventional biological samples can limit the discovery of robust biomarkers, making them less suitable for the study of long-term effects of environmental exposures on pregnancy outcomes. Conversely, hair is a highly stable structure that assimilates endogenous compounds and environmental compounds during growth. These accumulate in an ordered temporal manner as hair grows. Hair sampling is also non-invasive compared to the collection of other biological samples such as blood. As a result, a hair sample offers several potential advantages over other biospecimens, such as providing a metabolite profile that reflects environmental exposures over several months (41). In addition, our previous studies have found that the maternal hair metabolome can reflect differences between healthy pregnancies and complicated pregnancies, including fetal growth restriction (FGR) and GDM (42–44). Previously conducted hair metabolomic studies have used a case-control design with a relatively small sample size. To date, no study has investigated the maternal hair metabolome in a large cohort in an attempt to understand how external exposures may alter the metabolic profile in association with GDM.

The aim of this research was to comprehensively assess the relationship between air pollution, the maternal hair metabolome and GDM status from the pre-conception period through the three trimesters of pregnancy.

## Methods

### Study participants

**Figure 1:**
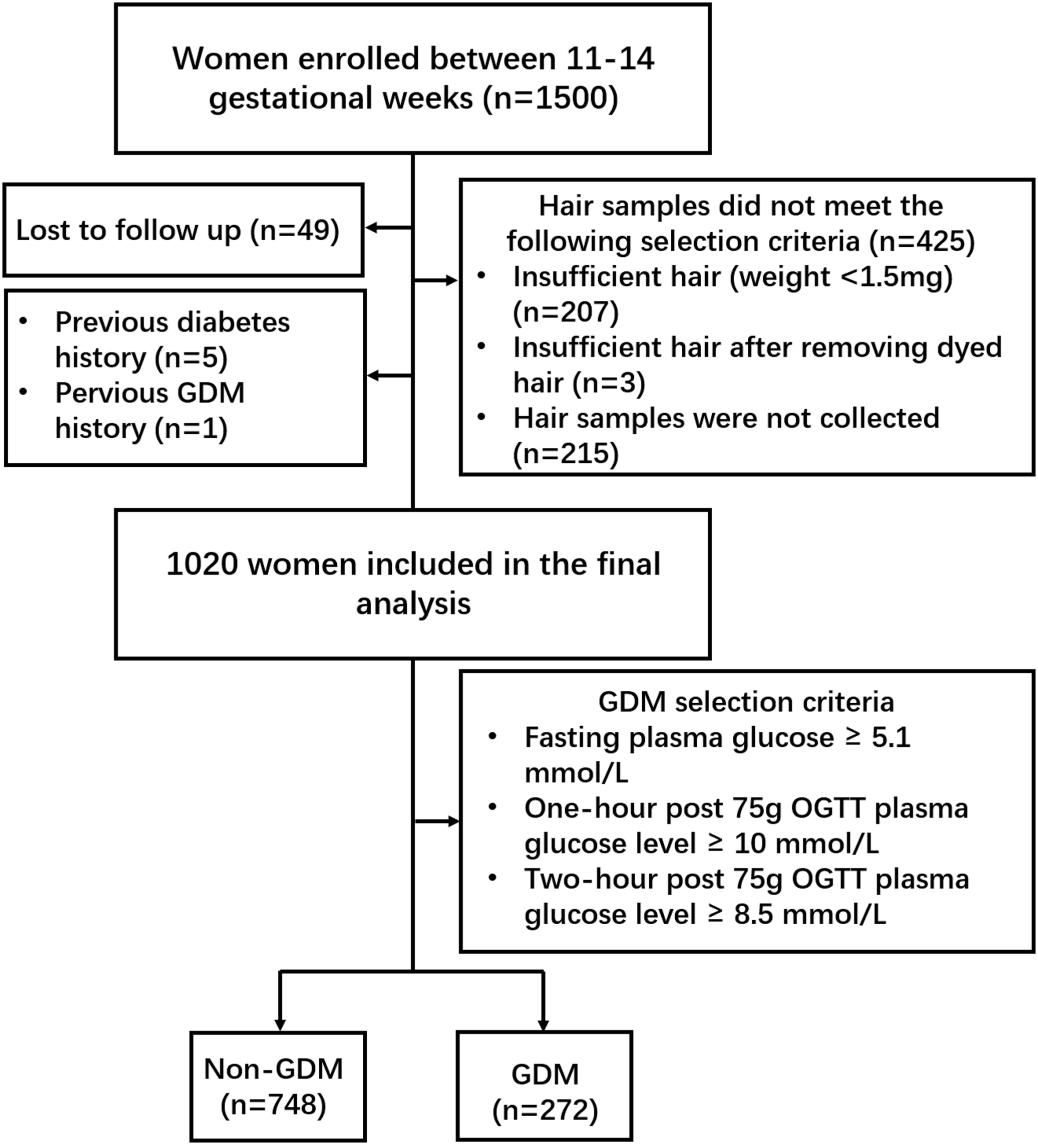
Flowchart of recruited participants. In total, 1500 pregnant women were enrolled between 11-14 gestational weeks. Forty-nine women were lost to follow up, five women with diabetes, one woman with a history of GDM, three women dyed hair, 207 women provided insufficient hair samples and 215 women did not provide a hair sample. A 75-g OGTT was conducted to diagnose GDM according to the IADPSG guidelines. Two hundred and seventy-two GDM women and 748 non-GDM women were included in the final analysis.

Women were recruited from the prospective, longitudinal CLIMB (Complex Lipids In Mothers and Babies) cohort, which has been described previously (45). Recruitment of the CLIMB cohort began in September 2015 and ended in November 2016. Among 17,382 eligible participants, 1500 pregnant women (response rate 8.6%) enrolled between 11-14 gestational weeks at the First Affiliated Hospital of Chongqing Medical University (FCQMU) and Chongqing Health Centre for Women and Children (CHC) (46). Women were included in the study if they were between 20–40 years of age and had a singleton pregnancy. Women were excluded from the study if they had a previous pregnancy with complications which resulted in delivery before 32 weeks. We also excluded women lost to follow up (n=49), women with diabetes (n=5), women with a history of GDM (n=1), those with insufficient hair samples (weight < 1.5 mg, n=207; dyed hair, n=3) and those who did not provide a hair sample (n=215) (**Figure 1**). A 75-g oral-glucose-tolerance test (OGTT) was conducted between 24 and 28 weeks of gestation to identify women with and without GDM. Diagnosis of GDM occurred according to the International Association of Diabetic Pregnancy Study Group (IADPSG) guidelines (fasting plasma glucose ≥ 5.1 mmol/L, one-hour post 75g OGTT plasma glucose level ≥ 10 mmol/L, or two-hour post 75g OGTT plasma glucose level ≥ 8.5 mmol/L) (47). This study was conducted in accordance with the principles in the Declaration of Helsinki. Ethical approval was granted by the Ethics committee of the Chongqing Medical University (No. 2014034), and written informed consent was obtained from all participants.

### Hair sample collection and preparation

Maternal hair strands (n=1020) were collected at 32-34 weeks of gestation. Hairs were cut 0.5 cm away from the scalp around the occipital area at the rear of the head and stored in aluminum foil at -20 °C. The hair segments were cut according to the method published by Delplancke et al. (48). The samples for the first segment from the scalp-end of the hair (0–3 cm) were enriched for growth that occurred in the early third trimester. The next 0.5 cm segment (3–3.5 cm) was discarded to avoid the overlap between the segments for the third and second trimester. The samples for the second segment (3.5-6.5 cm, second trimester), third segment (7-10 cm, first trimester), and fourth segment (10.5-13.5 cm, pre-conception) were collected; (0.5 cm between each segment was discarded). All four hair segments were used for our final analysis.

All hair segments were washed and metabolites extracted using the methods published by Sulek et al. (42). Dried hair extracts were stored at −80°C prior to chemical derivatization. Firstly, hair segments were washed with Milli-Q (MQ) water and methanol twice. The hair segments were put inside glass vials and weighed (range was between 1.5 to 5.5 mg). Alkaline hydrolysis was performed by adding 400 µl of potassium hydroxide (1M) and 20 µL of the internal standard mix (2,3,3,3-d4-alanine (10 mM); 2,3,4,5,6-d5-phenylalanine (10 mM); 3,3-d2-tyrosine (2 mM)) and incubated at 54°C for 18 h. Then, the hair extracts were neutralized to pH 7 by the addition of 67 µl of sulphuric acid (3M). To remove salts and proteins, 1 ml of methanol was added and the sample centrifuged at 4000 rpm for 5 min. Portions (350 µL) of the supernatant were then transferred to three microfuge tubes. The quality control (QC) samples were prepared by pooling the remaining supernatant (approx. 100 µL) from each sample into one 15 ml centrifuge tube, mixing, and then dividing into 350 µL aliquots. All the extracts and QCs were evaporated to dryness using a speedvac (CentriVap Concentrator 230V 50Hz, Cold Trap −105°C Models, Labconco, USA) for 8 h and stored at −80°C prior to derivatization.

### Methyl chloroformate derivatization

All prepared samples were chemically derivatized by methyl chloroformate prior to GC-MS analysis, as described previously (49, 50). The dried hair extracts were resuspended in 200 μl of sodium hydroxide (1M). Then samples were transferred to a salinized glass tube and both 167 µl methanol and 34 µl pyridine were added. Derivatization was initiated by adding 20 µL MCF followed by mixing for 30 seconds and this step was repeated twice. MCF derivatives were partitioned by adding 400 µL chloroform and mixing for 10 seconds. Subsequently, 400 µL of sodium bicarbonate (50 mM) was added and mixed for an additional 10 seconds. The samples were then centrifuged at 1500 rpm for 5 minutes before the upper aqueous layer was discarded. Finally, the remaining water was eliminated by adding anhydrous sodium sulphate and the derivatized samples were transferred to GC vials for GC-MS analysis.

### Gas chromatography-mass spectrometry analysis

The hair MCF derivatives were analyzed using an Agilent GC7890B system linked to a MSD5977A mass selective detector (EI) set at 70 eV. The GC column used for metabolite analysis was a ZB-1701 GC capillary column (30 m x 250 μm id x 0.15 μm with 5 m guard column, Phenomenex). The GC analysis parameters were as previously described (49, 51). All samples were introduced *via* pulsed splitless injection with the inlet temperature at 290°C. A constant helium gas flow rate of 1 mL/min was used. The GC-oven was first held at 45°C for 2 min, and then the temperature was elevated with a gradient of 9°C/min to 180°C and was held for 5 min. The temperature was then raised at 40°C/min to 220°C and was held for 5 min. Then the temperature was elevated at 40°C/min to 240°C and was held for 11.5 min. Finally, the temperature was raised at 40°C/min to 280°C and was held for 7 min. The mass spectrometer was run under scan mode with a speed of 3.12 scans/sec with a mass range between 38-550 amu. The solvent delay ended after 5 min. The auxiliary temperature was set to 250°C, the ion source temperature was set to 230°C, and the quadrupole temperature was 150°C (50).

### Data extraction and normalization

Raw GC-MS chromatograms were deconvoluted using Automated Mass Spectral Deconvolution and Identification System (AMDIS) software and metabolites were identified using our in-house MCF mass spectral library of 461 compounds and National Institute of Standards and Technology (NIST14) mass spectral library. The criteria for metabolite identification included a match to the MS spectrum of the library compound of >75% and within a 1 min time window of its respective chromatographic retention time. The relative concentrations of the identified metabolites were calculated using MassOmics R-based software (52) that selected the peak height of the most abundant ion within an expected retention time bin. After manual removal of contaminants by comparison with the blank samples, GC-MS data were first normalised by the multiple internal standards (2,3,3,3-d4-alanine, 2,3,4,5,6-d5-phenylalanine, 3,3-d2-tyrosine) and initial batch correction was performed using median centring according to metabolite levels in the QC samples. Lastly, the data were normalised by hair biomass.

### Data on air pollution exposure

**Figure 2:**
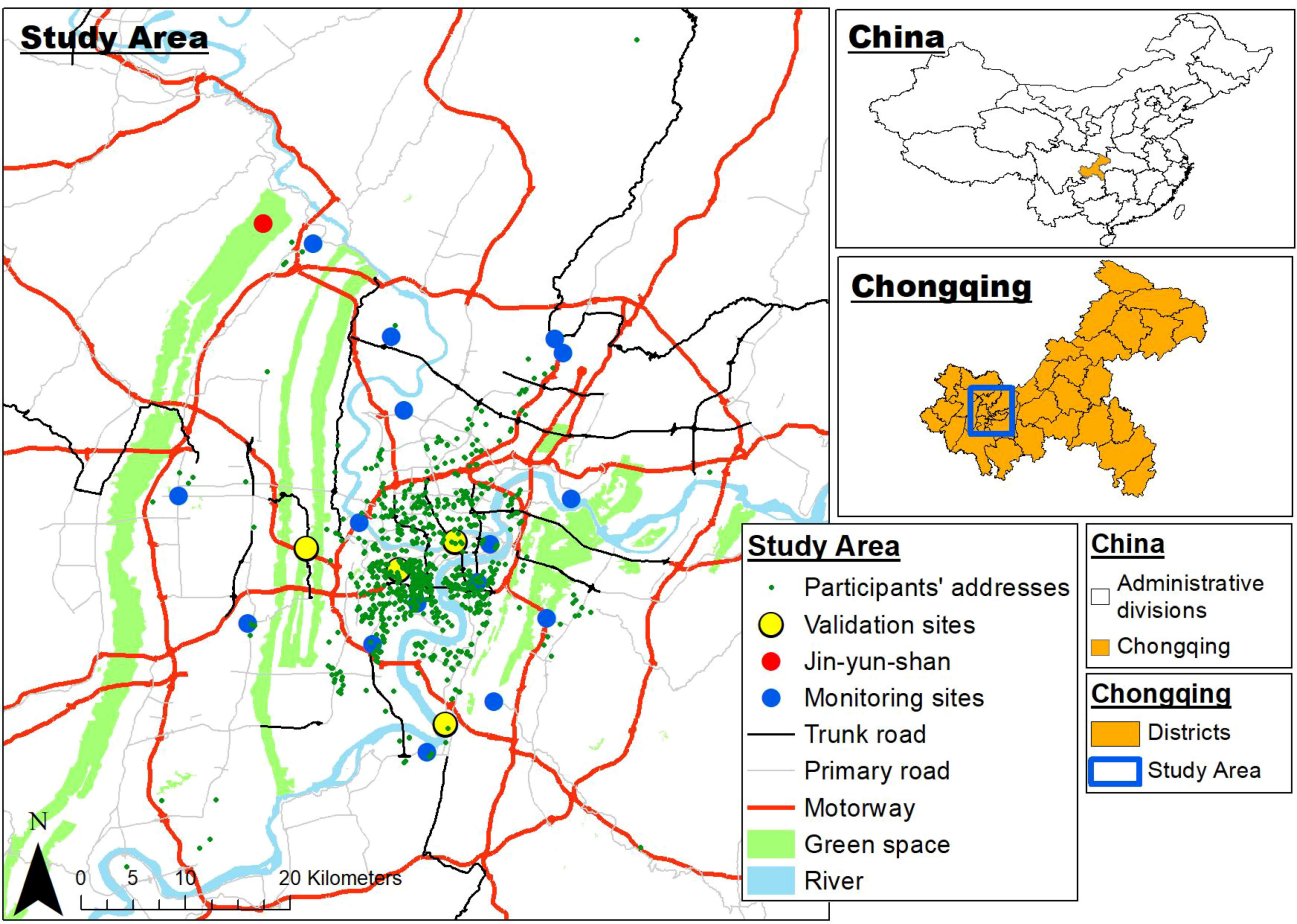
Map of Chongqing showing the location of the monitoring sites and participants’ residential locations.

The concentrations of particulate matter less than 2.5μm in diameter (PM_2.5_), less than 10μm in diameter (PM_10_), sulfur dioxide (SO_2_), nitrogen dioxide (NO_2_), ozone (O_3_), and carbon monoxide (CO) are indicators of air quality. Air pollution measurements were obtained from the reports published by the Chongqing Ecological Protection Bureau (53) between 1 January 2015 and 31 December 2017. Data were collected from twenty-one air quality monitoring stations in the main urban area of Chongqing (**Figure 2**). Monitoring sites were given a site ID number and classified as traffic sites if they were <100m from a major road (motorway, trunk road, primary road), and background sites if they were >100m away from a major road (**Supplementary Table 1**). In this study, we have used two methods, namely proximal and land use models, to estimate the amount of maternal exposure during pregnancy. With regards to the proximal model, each pregnant woman was linked to the nearest air quality monitoring station data, according to her residential address. Exposures were calculated as daily concentrations averaged over each of the trimesters. Furthermore, Harper et al. (54) developed spatiotemporal land use regression (LUR) models for PM_2.5_ and NO_2_ in Chongqing, China, and used the models to estimate PM_2.5_ and NO_2_ exposure for the participants in the CLIMB study. The accuracy of LUR model in estimating PM_2.5_ and NO_2_ exposures was assessed by the comparing measured and predicted pollutant levels using leave-one-out-cross validation (LOOCV), as displayed in **Supplementary Table 2**. Therefore, in additional to measured concentrations from the nearest monitoring site, we used the LUR model to estimate the PM_2.5_ and NO_2_ exposure of our study population.

### Statistical analysis

The distribution of maternal clinical characteristics and air exposure data were evaluated using the quantile-quantile (Q-Q) plot and Shapiro-Wilk Normality Test. Student’s T-tests were conducted for normally distributed data, while Mann-Whitney tests were used for non-normally distributed data. All metabolite intensities below the detectable threshold were replaced with 0.5 times the smallest non-zero value for the relevant metabolite and subsequently log transformed to produce a Gaussian distribution. The transformed metabolite profiles were used to perform Uniform Manifold Approximation and Projection (UMAP) dimensionality reduction and different clusters were annotated using the R package UMAP v0.2.8.0 (55). Logistic regression models were created for the four periods, including the confounding factors BMI and age. Metabolites were considered significant if they had a *p*-value and *q*-value less than 0.05. A linear mixed model was applied to assess the relationship between GDM and the hair metabolite levels across the four timepoints, using the lme4 R package (56). Hair metabolites were entered into the linear mixed model as the response variable; GDM and hair segment were entered as fixed effects; and experimental batch and individual were entered as random effects. Unadjusted models and models adjusted for major confounding variables as additional fixed effects were established. Confounding variables considered when assessing the relationship between GDM and hair metabolite levels were maternal age and maternal BMI relevant to the timepoint of each hair segment. Metabolites concentrations that were significantly different between GDM and non-GDM groups were based on the likelihood ratio test comparing a null model without GDM as a predictor to one with both a main effect of GDM, and an interaction between GDM and hair segment (indicating differential changes over time). False discovery rates (*q*-values) were used to account for multiple comparisons, using the R package “qvalue” (57). The parameter π_0_ was set to the conservative value of 1 rather than estimated. Metabolites were considered significant if they had a *q*-value less than 0.05. Significant results were then assessed for robustness by trimming the top 0.5% and bottom 0.5% of measurements. The Odds ratios (ORs) were estimated by logistic regression models to analyzed the association between air pollution exposures (increase per interquartile range (IQR)) and GDM risk from pre-conception to third trimester periods either with or without adjustment for maternal age and BMI. To investigate associations between the air pollution exposure data and the metabolites found to be significantly associated with GDM, mixed models were fitted with the GDM associated metabolite as the response, and the pollutant as the predictor, controlling for the effect of segment, batch, and individual effects. The set of *p*-values produced across pollutants and GDM associated metabolites underwent the *q*-value procedure, and associations were considered statistically significant if they had a *q*-value less than 0.05.

## Results

### Participant characteristics

The prevalence of GDM in our cohort (27.1%) was slightly higher than in the general population of China (ranging from 17.6% to 24.24%) (58, 59). In total, 1020 CLIMB participants were included in the study; 272 women were diagnosed with GDM according to the IADPSG guidelines and 748 women were included in the non-GDM group. Clinical characteristics for all participants are listed in **Table 1**. As anticipated, the fasting, one-hour, and two-hour blood glucose levels following the 75g OGTT were statistically different between GDM and control groups (*p* < 0.001). Compared to women in the control group, participants with GDM had a significantly higher age and BMI (in pre-conception and into the third trimester, *p* < 0.001). Moreover, women in the GDM group tended to deliver earlier than women in the control group (*p* = 0.027). Education level, blood pressure, placental weight, and birth weight exhibited no significant differences between the GDM and control groups.

**Table 1:** Clinical characteristics of the study participants.

	GDMn = 272	Non-GDMn = 748	*p*-value
Maternal Age, years	29 (27, 32)	28 (26, 30)	<0.001^a^
Maternal education, years	16 (15,16)	16 (15,16)	0.667^a^
Pre-pregnant BMI, kg/m^2^	21.6 (19.8, 23.5)	20.7 (19.1, 22.6)	<0.001^a^
First-trimester BMI, kg/m^2^	22.0 (20.0, 24.0)	20.9 (19.3, 22.8)	<0.001^a^
Second-trimester BMI, kg/m^2^	23.8 (22.2, 26.0)	23.1 (21.5, 25.2)	<0.001^a^
Third -trimester BMI, kg/m^2^	25.4 (23.4, 27.4)	24.6 (22.7, 26.6)	<0.001^a^
OGTT_fasting, mmol/L	5.1 (4.7,5.2)	4.6 (4.4,4.7)	<0.001^a^
OGTT_1h, mmol/L	9.5 (8.6,10.4)	7.2 (6.4,8.3)	<0.001^a^
OGTT_2h, mmol/L	8.5 (7.5,9.1)	6.7 (6.0,7.4)	<0.001^a^
First-trimester Systolic blood pressure, mmHg	114 (108,121)	111 (106,120)	0.140^a^
First-trimester Diastolic blood pressure, mmHg	71 (66,77)	70 (65,74)	0.102^a^
Second-trimester Systolic blood pressure, mmHg	115 (109,120)	114 (108,121)	0.077^a^
Second-trimester Diastolic blood pressure, mmHg	70 (68,75)	70 (67,72)	0.081^a^
Third -trimester Systolic blood pressure, mmHg	117 (109,121)	115 (108,121)	0.090^a^
Third -trimester Diastolic blood pressure, mmHg	70 (66,74)	70 (65,73)	0.156^a^
Gestational age, weeks	39 (38,40)	39 (39,40)	0.027^a^
Placental weight, g	560 (510,600)	550 (510,600)	0.299^a^
Infant birth weight, g	3321 ± 427	3331 ± 395	0.741^b^

Values are means ± SD or median (IQR).

a p-value from Mann-Whitney test,

b p-value from Student T-test.

### Hair metabolites and GDM status

**Figure 3:**
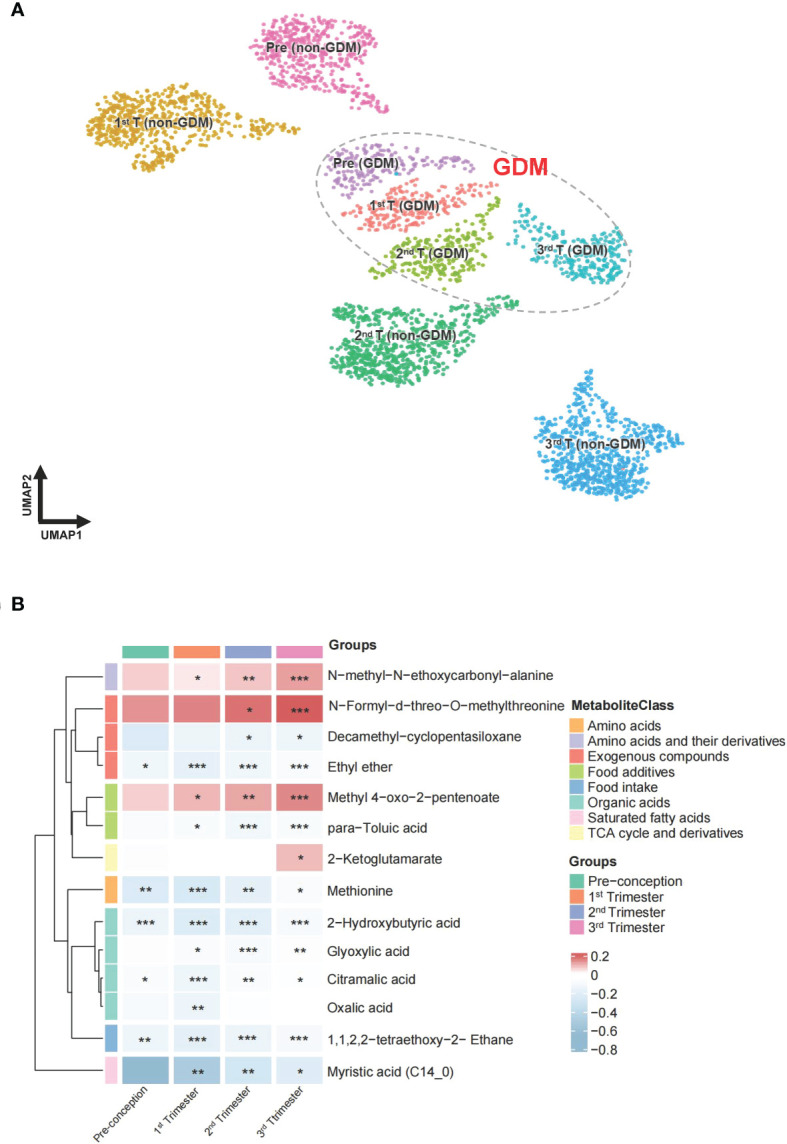
UMAP projection and heatmap of the metabolites across pregnancy. **(A)** UMAP clustering of all participants labelled and colored by GDM status and different periods of gestation. Groupings include pre-conception GDM [pre (GDM)], first trimester GDM [1^st^ T (GDM)], second trimester GDM [2^nd^ T (GDM)], third trimester GDM [3^rd^ T (GDM)], pre-conception non-GDM [pre (non-GDM)], first trimester non-GDM [1^st^ T (non-GDM)], second trimester non-GDM [2^nd^ T (non-GDM)], and third trimester non-GDM (3^rd^ T (non-GDM)). **(B)** The ratio of fourteen metabolite levels significantly different between GDM case and control groups. Red color indicates higher metabolite levels in the GDM group than the control group, while blue color indicates lower metabolite levels in the GDM group than the control group. Metabolites with both *p*-value and *q*-value less than 0.05 in the logistic regression adjusted for age and BMI are marked with *p-value and *q*-value less than 0.01 are marked with **p-value and *q*-value less than 0.001 are marked with ***.

**Figure 4:**
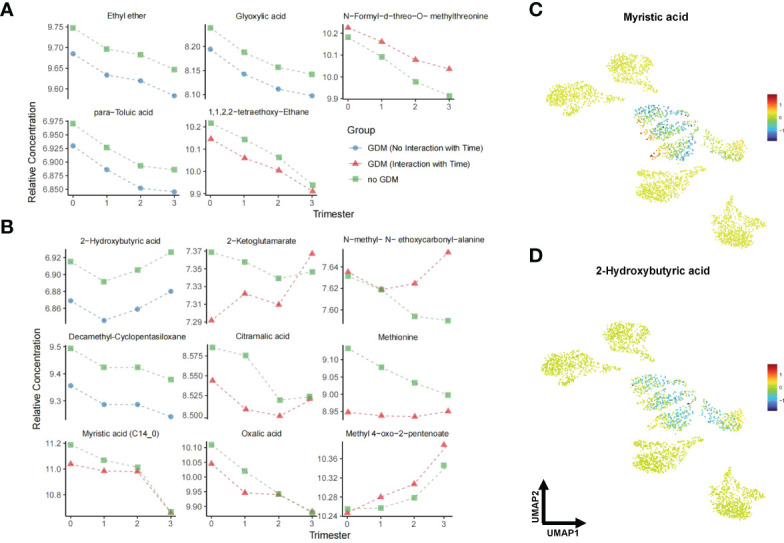
Line plots and UMAP of GDM-associated metabolites across pregnancy. **(A)** shows unadjusted models while **(B)** shows the results of linear mixed models adjusted for maternal age and BMI. Triangles represent the fitted effects in GDM maternal hair and squares represent non-GDM. Blue denotes a consistent effect across gestation in the GDM group (no significant interaction with time), while red corresponds to a time-specific effect. **(C, D)** UMAP representation of myristic acid and 2-hydroxybutyric acid from data in Figure 3 **(A)**. Red dots indicate higher metabolite levels in GDM women compared with the non-GDM women, while blue dots indicate lower levels. Data are visualized after log transformation.

A total of 276 metabolites were identified in the hair of the participants. Information on all identified metabolites is listed in **Supplementary Tables 3–7**. All identified metabolites were integrated into a UMAP. Participant clusters were generated using low resolution nearest-neighbor clustering and these were divided into eight clusters according to GDM and non-GDM groups across four time points. The UMAP scatter plot of dimensional reduction colored by participants’ identity is shown in **Figure 3** **(A)**. Clusters were positioned approximately from pre-conception to the third trimester in a diagonal south-eastward direction. GDM groups were clustered in the middle while non-GDM groups were scattered around. The common significant metabolites in the logistic regression and linear mixed model were shortlisted to form a final list of metabolites significantly associated with GDM. After adjustment for multiple comparisons, fourteen metabolites were found to be significantly different (*q* < 0.05) between GDM cases and non-GDM controls at one timepoint or more from pre-conception to the third trimester of pregnancy and robust in adjusted for confounders (maternal age and BMI) (**Table 2**). In **Figure 3B**, the heatmap demonstrates the ratios of the fourteen significant metabolites between cases and controls from pre-conception through each of the trimesters. In pre-conception, only five hair metabolite levels were significantly reduced in the GDM group compared to the non-GDM group. Whereas, ten hair metabolites were significantly different between the two groups across all trimesters, including some amino acids and derivatives, fatty acids, organic acids, food additives, and exogenous compounds. 2-hydroxybutyric acid, methionine and myristic acid showed the most substantial ratio changes. Furthermore, nine of these fourteen metabolites demonstrated a significant interaction with the hair segment tested (timepoint 0, 1, 2, or 3) – indicating that there were longitudinal differences in the concentrations of these metabolites and their relationship with GDM (**Figure 4**). Of these metabolites, six showed the largest differences from pre-conception, with differences reducing in later trimesters. As displayed in **Figures 4A, B**, two of the fourteen metabolites were consistently higher in GDM participants compared to controls across all four timepoints; ten were consistently lower in GDM participants across the timepoints (i.e. 2-hydroxybutyric acid and myristic acid); and two metabolites had relationships with GDM which changed direction between timepoints (i.e. 2-ketoglutamarate, and methyl 4-oxo-2-pentenoate were lower in GDM participants in the pre-conception hair segments but were higher in GDM participants in the third trimester hair segments). Interestingly, both 2-hydroxybutyric acid and myristic acid displayed reduced levels in GDM subclusters, as highlighted in the UMAP (**Figures 4C, D**).

**Table 2:** Metabolites with significant and robust differences between GDM cases and non-GDM controls in logistic regression and linear mixed models.

Metabolites	Logistic regression (*q*-value)				Linear mixed model			
	Pre-conception	First trimester	Second trimester	Third trimester	*p-*value	*p*-value(adjusted)	*q*-value	*q*-value(adjusted)
Oxalic acid	0.110	0.002	0.181	0.056	0.003	<0.001	0.041	0.012
N-Formyl-d-threo-O-methylthreonine	0.197	0.012	0.003	<0.001	0.001	0.049	0.027	0.321
Methyl 4-oxo-2-pentenoate	0.122	0.037	0.004	<0.001	0.026	0.004	0.221	0.049
Decamethyl-cyclopentasiloxane	0.110	0.053	0.046	0.037	<0.001	<0.001	<0.001	0.009
Ethyl ether	0.017	<0.001	<0.001	0.001	0.001	<0.001*	0.026	0.009*
2-Hydroxybutyric acid	0.001	<0.001	<0.001	<0.001	0.002	0.001	0.030	0.020
Citramalic acid	0.014	<0.001	0.002	0.011	<0.001	<0.001	0.016	0.008
para-Toluic acid	0.365	0.034	<0.001	<0.001	0.001	0.010	0.023	0.109
Glyoxylic acid	0.417	0.014	<0.001	0.007	<0.001	0.002*	0.011	0.025*
2-ketoglutamarate	0.289	0.270	0.416	0.027	<0.001	0.001	0.016	0.017
1,1,2,2-tetraethoxy-2- Ethane	0.004	<0.001	<0.001	<0.001	<0.001	<0.001*	0.016	0.008*
N-methyl-N-ethoxycarbonyl-alanine	0.266	0.184	0.018	<0.001	0.001	0.001	0.019	0.016
Myristic acid (C14_0)	0.132	0.004	0.010	0.014	0.006	0.002	0.085	0.025
Methionine	0.009	<0.001	0.005	0.024	0.001	<0.001	0.019	0.009

Values marked with a *correspond to p > 0.05 after trimming of extreme values.

### Air pollutions and GDM

**Figure 5:**
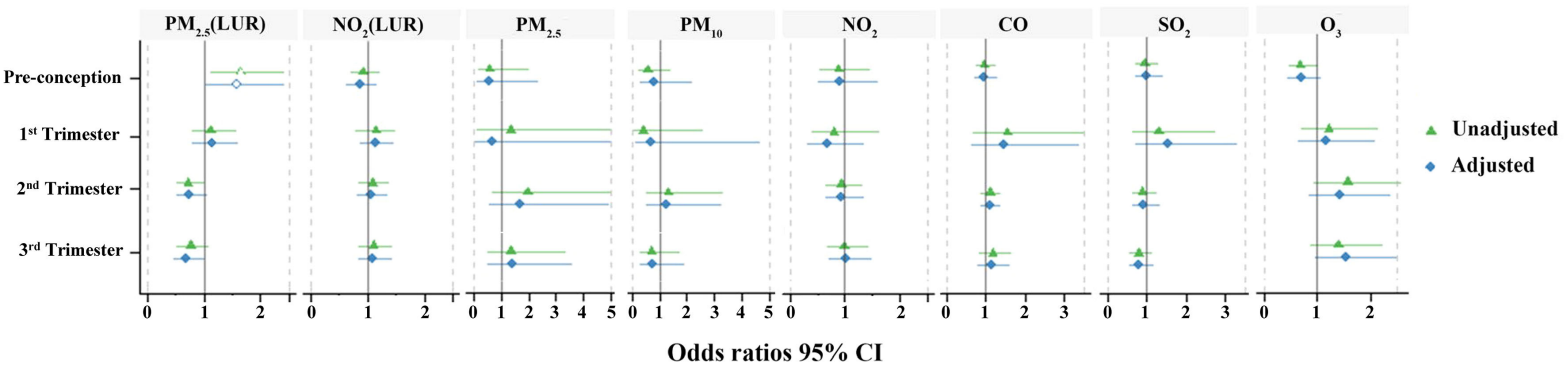
Odds ratios between air pollution exposures and GDM occurrence. Green indicates the unadjusted odds ratio (95% CI); blue indicates the adjusted odds ratio (95% CI). Hollow shapes represent statistical significance, while solid shapes are non-significant.

The daily average calculated exposure concentrations of air pollution for each timepoint based on the proximal model are listed in **Table 3**. **Table 4** describes the exposure estimates for PM_2.5_ and NO_2_ using the LUR model. The estimates of PM_2.5_ and NO_2_ according to the LUR model were higher in the GDM group during pre-conception and early pregnancy, respectively (*p* < 0.05). **Figure 5** demonstrates the association between air pollution and GDM risk across four periods. After adjusting for maternal age and BMI, we only found that exposure to PM_2.5_ (LUR) within the pre-conception (OR=1.584, 95% CI: 1.032-2.432, *p*=0.035) was significantly associated with GDM occurrence. This result was consistent with PM_2.5_ estimated by the LUR model, which was higher in the GDM group during pre-conception.

**Table 3:** Daily average concentrations exposure of the pollutants using the proximal model.

Pollutant (µg/m³)	Pre-conception		First trimester		Second trimester		Third trimester	
	Non-GDM	GDM	Non-GDM	GDM	Non-GDM	GDM	Non-GDM	GDM
**PM _2.5_**								
Min	22.6	16.4	15.7	22.2	20.8	22.4	20.8	23.3
Median (IQR)	47.8 (40.9, 56.5)	46.7 (39.8, 57.1)	51.8 (40.6, 58.6)	49.0 (40.1, 61.4)	55.3 (43.2, 64.6)	52.3 (43.5, 63.4)	50.8 (42.0, 62.2)	49.4 (41.9, 59.5)
Max	80.1	76.7	82.5	116.5	81.1	83.2	79.7	76.9
**PM _10_**								
Min	34.0	24.9	19.8	34.4	32.8	34.3	36.8	38.7
Median (IQR)	80.1 (65.8,91.1)	76.6 (63.3, 91.1)	79.4 (64.9, 91.8)	76.5 (64.4, 91.1)	83.1 (66.2, 93.7)	81 (66.7, 91.5)	80.6 (66.8, 93.3)	77.9 (65.5, 91.0)
Max	116.3	114.8	117.0	116.5	118.3	118.3	118.1	117.5
**SO_2_**								
Min	7.4	2.8	4.5	7.4	5.4	7.2	6.7	7.3
Median (IQR)	12.1 (10.8, 14.5)	12.5 (11.1, 14.8)	12.0 (10.6, 14.6)	12.4 (10.9, 14.9)	13.2 (10.3, 15.1)	12.6 (9.9, 15.2)	12.1 (9.8, 14.6)	11.5 (9.6, 14.3)
Max	27.2	24.8	25.1	25.6	26.2	25.8	25.8	23.9
**NO_2_**								
Min	10.1	10.1	7.9	10.1	10.4	12.9	11.4	12.3
Median (IQR)	47.8 (33.3, 61.9)	42.5 (30.0, 60.1) *	47.7 (33.5,61.3)	43.4 (30.5, 61.6)	51.0 (36.8, 63.9)	47.2 (35.0, 63.1)	51.1 (35.8, 65.8)	46.8 (32.8, 64.0)
Max	74.5	75.0	74.6	74.9	75.4	74.6	75.2	74.6
**CO**								
Min	0.5	0.2	0.1	0.5	0.5	0.5	0.5	0.5
Median (IQR)	0.9 (0.8, 1.0)	0.9 (0.8, 1.1)	1.0 (0.8, 1.1)	1.0 (0.86, 1.1)	1.0 (0.9, 1.1)	0.9 (0.8, 1.1)	1.0 (0.8, 1.1)	0.9 (0.8,1.1)
Max	1.5	1.6	1.7	1.6	1.6	1.6	1.6	1.6
**O_3_**								
Min	6.4	6.4	6.4	6.5	6.5	6.5	6.6	6.6
Median (IQR)	39.4 (26.5,53.8)	39.1 (24.2, 54.6)	38.4 (20.5, 53.3)	37.2 (19.8, 53.0)	29.6 (14.8, 50.8)	34.0 (18.3, 51.3)	39.2 (20.8, 54.0)	46.2 (26.8, 55.4) *
Max	112.5	112.4	115.1	114.0	115.0	115.2	115.2	115.0

*p-value less than 0.05 between GDM and non-GDM.

Exposures were calculated as daily concentrations averaged over each timepoint (pre-conception – third trimester).

**Table 4:** Exposure estimate data of PM_2.5_ and NO_2_ using the LUR model.

Pollutant (µg/m³)	Pre-conception		First trimester		Second trimester		Third trimester	
	Non-GDM	GDM	Non-GDM	GDM	Non-GDM	GDM	Non-GDM	GDM
**PM _2.5_**								
Min	39.2	38.4	37.5	38.3	38.5	38.5	37.6	37.0
Median (IQR)	53.1 (44.0, 62.5)	55.9 (45.0, 65.6) **	52.0 (43.9, 61.1)	53.5 (44.0, 63.5)	58.6 (47.6, 67.1)	56.6 (46.4, 66.0)	61.8 (47.1, 75.8)	59.9 (46.8, 69.7)
Max	80.3	79.5	82.4	80.0	89.1	90.0	94.7	93.6
**NO_2_**								
Min	26.1	25.9	28.4	29.1	32.4	31.1	23.5	20.6
Mean ± SD	49.6 ± 6.2	50.5 ± 6.9	48.7 ± 6.4	49.7 ± 6.6 *	51.0 ± 6.3	50.5 ± 6.3	51.7 ± 6.8	51.5 ± 7.0
Max	66.6	67.1	69.3	67.3	68.4	70.4	75.1	70.7

*p-value less than 0.05 between GDM and non-GDM.

**p-value less than 0.01 between GDM and non-GDM.

### Air pollutions and hair metabolites

**Figure 6:**
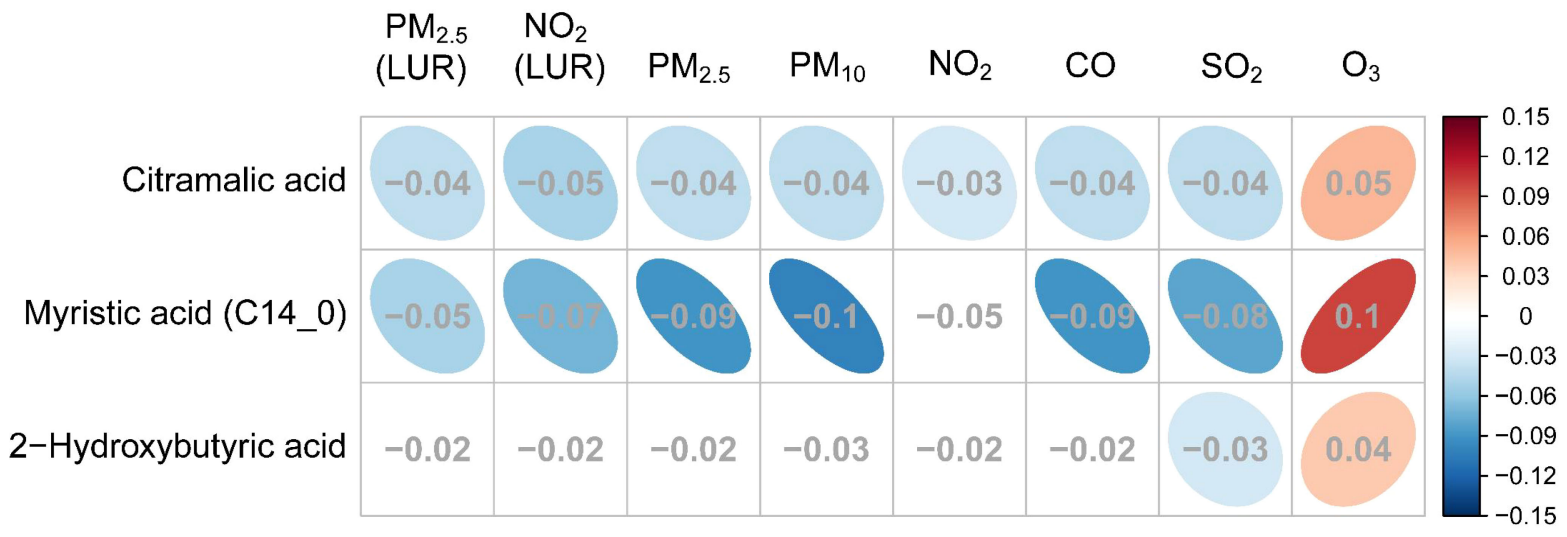
The association between GDM related metabolites and specific air pollutants. The grey numbers are standardized regression coefficients that show the effect of one standard derivation (SD) pollutant change on the standardized log metabolite concentration. The red right-handed ellipses indicate positive relationships between metabolites and air pollutants, while the blue left-handed ellipses indicate negative relationships. Only the significant associations (*q*-values < 0.05) between metabolites and pollutants are displayed by ellipses.

The relationships between GDM-associated hair metabolites and specific pollutants (including daily average concentrations of PM_2.5_, PM_10_, SO_2_, NO_2_, O_3_, CO and the exposure estimate data of PM_2.5_ and NO_2_) are illustrated in **Figure 6**. Three of the fourteen hair metabolites (2-hydroxybutyric acid, citramalic acid, and myristic acid) that showed a significant association with GDM were also significantly associated with some, or all of the air pollutant measures (*q* < 0.05, **Table 5**). Each of these were negatively associated with daily average concentrations of PM_2.5_, PM_10_, SO_2_, NO_2_, CO and the exposure estimate of PM_2.5_ and NO_2,_ and positively associated with O_3_.

**Table 5:** *q*-values for metabolites with significant association with one or more pollutants.

*q*-value	PM_2.5_(LUR)	NO_2_(LUR)	PM_2.5_	PM_10_	NO_2_	SO_2_	CO	O_3_
Citramalic acid	**<0.001**	**<0.001**	**<0.001**	**<0.001**	**0.031**	**<0.001**	**<0.001**	**<0.001**
Myristic acid (C14_0)	**<0.001**	**<0.001**	**<0.001**	**<0.001**	0.275	**<0.001**	**<0.001**	**<0.001**
2-Hydroxybutyric acid	0.089	0.461	0.072	0.061	0.487	**0.002**	0.092	**0.002**

The bold values means q-value less than 0.05.

Standardized coefficients (showing the effect of one standard derivation pollutant change on the standardized log metabolite concentration) for PM_2.5_ and NO_2_ exposure estimated by the proximal and LUR models displayed a similar correlation, three metabolites were all negatively associated with PM_2.5_ and NO_2_. However, only citramalic acid was significantly associated with NO_2_ exposures estimated by the proximal model, but myristic acid and citramalic acid were remarkable in the LUR model, indicating that exposure estimation by the LUR model showed more statistical significance than the proximal model.

## Discussion

To our knowledge, this is the first study to investigate associations between environmental exposures, the hair metabolome, and GDM longitudinally from pre-conception through to the third trimester. The main focus of hair metabolomic research to date has been the identification of metabolic biomarkers for the prediction of pregnancy complications (42–44). Our results demonstrated that the maternal hair metabolome was altered in response to endogenous and exogenous exposures, prior to, and throughout pregnancy. Moreover, exposure to PM_2.5_ during pre-conception may increase the risk of GDM. The levels of 2-hydroxybutyric acid, citramalic acid, and myristic acid in the hair of GDM women were negatively associated with the air pollutant levels. Our findings suggest that the hair metabolome changes in response to maternal and environmental perturbations with substantial potential to estimate exposure risk factors and better understand the underlying GDM pathophysiology.

### Pre-conception exposure to air pollution may increase the risk of GDM

Air pollution has become a major public health issue in developed and developing countries (1). Particulate matter (PM) is composed of complex chemical constituents from a variety of sources, such as elemental carbon, metals, and organic chemicals derived from coal combustion, biomass burning, vehicle emissions, dust, and industrial sources (60). We have published a LUR model that was developed on a daily basis incorporating measurement data, temporal variables on meteorology, and spatial variables produced using a geographical information system (54). This spatiotemporal model demonstrated better performance than the proximal model in discriminating GDM and non-GDM groups from pre-conception to the first trimester. Our results demonstrated that the estimates of PM_2.5_ and NO_2_ by the LUR model were higher in the GDM group during pre-conception and in early pregnancy, respectively. Meanwhile, the calculated daily average exposure to PM_2.5_ and NO_2_ based on the proximal model did not show any differences from pre-conception to third trimester. Consistently, evidence from our study also suggested that PM_2.5_ exposure during pre-conception increased the risk of GDM. Similar to our results, Zhang et al. found that higher exposure to PM_2.5_ within three months before pregnancy was associated with increased GDM risk, as well as elevated fasting glucose levels (61). Another study in China showed that pre-conception PM_2.5_ and PM_10_ exposure was associated with a higher risk of developing GDM (62). In a case-control study in Taiwan, researchers found that higher pre-conception and pregnancy exposures to PM_2.5_ for mothers were associated with a significantly elevated risk of GDM (63). Yu et al. indicated that exposure to PM_2.5_ in the second trimester of pregnancy was associated with an increased risk of GDM (64). A pregnancy cohort in Southern California showed that pre-conception NO_2_ was associated with an increased risk of GDM, and first trimester NO_2_ was weakly associated with GDM (65). The majority of studies found that maternal exposure to PM_2.5_ and NO_2_ was associated with a significantly elevated risk of GDM. However, the results of recent meta-analyses indicated that increased exposure to PM_2.5_ was not statistically related to the incidence of GDM (30, 66). It is worth noting that inconsistence among these studies may reflect intrinsic dissimilarities in exposure assessment, outcome definition, effect estimation models and adjusted variables, which may contribute to the heterogeneity (31). Overall, these results suggest that exposure to higher levels of PM_2.5_ and NO_2_ during early pregnancy may increase susceptibility to GDM development.

### The potential relationship between air pollution and GDM

It remains unclear how air pollution and its components are associated with GDM. Previous studies suggested that oxidative stress and inflammation may play important roles in the development of GDM. Oxidative stress is a well-recognised risk factor for insulin resistance. Several studies have shown unequivocally that oxidative stress precedes insulin resistance (67–69). An epidemiological study showed that air pollution exposure could increase markers for oxidative stress among pregnant women (70). Particulate matter (PM) is enriched with metals and organic chemicals and has been thought to initiate toxic effects and induce oxidative damage (71, 72). For instance, PM_2.5_ alters levels of oxidative stress biomarkers in mice including glutathione peroxidase and malonic dialdehyde (73). Research has identified oxidative stress as one potential feature underlying the toxic effect of air pollution, which activates NF-κB translocation into the nucleus and causes an inflammatory response and cytokine production (74–77). Meanwhile, air pollution can produce pro-inflammatory mediators, including highly sensitive C-Reactive Protein, TNF-α, IL-1β, IL-6 and IL-8, resulting in local or systemic inflammation (8, 78, 79). Increased inflammatory response plays an important role in the development of GDM (80). Moreover, It has been argued that NO_2_ can cause similar inflammatory responses to those of PM (81). Both experimental and epidemiological studies indicate that air pollution may induce oxidative damage and trigger the release of inflammatory cytokines, subsequently contributing to insulin resistance, which consequently promotes the development of GDM.

### The association of air pollutants and hair metabolites in GDM

The metabolome reflects phenotypic changes in response to physiological status and environmental stimuli. There were three metabolites, myristic acid, 2-hydroxybutyric acid, and citramalic acid, found in significantly lower levels in the hair samples of women with GDM that were negatively associated with PM_2.5_, PM_10_, SO_2_, and CO. Of these three metabolites, myristic acid (14:0) levels were observed at lower levels in the hair samples of our GDM participants from the first trimester to the third trimester. Cumulative evidence has shown that fatty acids with different degrees of saturation affect insulin sensitivity and lipid/glucose metabolism differently. Saturated fatty acids are generally more likely to cause insulin resistance *via* inflammatory processes, while unsaturated fatty acids ameliorate the pathology associated with diabetes (82, 83). On the contrary, there are several studies showed that myristic acid has been inversely associated with a higher risk of type 2 diabetes (84–87). Interestingly, myristic acid has been demonstrated to increase glucose uptake in C2C12 skeletal muscle cells and reduce hyperglycaemia and insulin resistance in spontaneously diabetic Nagoya–Shibata–Yasuda (NSY) mice (88–90). However, the detailed effects of myristic acid on hyperglycaemia and insulin resistance have not been thoroughly investigated. It has been shown that the activity of delta-6(Δ6) desaturase (D6D) can be increased by myristic acid in cultured rat hepatocytes (91). Delta-6 desaturase, encoded by the fatty acid desaturase 2 (FADS2) gene, is the rate-limiting enzyme for the conversion of α-linolenic acid (ALA) to eicosapentaenoic acid (EPA) and docosahexaenoic acid (DHA) (92). Meanwhile, myristic acid has been reported to elevate EPA and DHA levels in human and rat plasma (93–95). EPA and DHA are long-chain polyunsaturated fatty acids (PUFAs), which have been found to be inversely associated with IL-6 and TNF-α concentrations and positively associated with the concentrations of the anti-inflammatory cytokines IL-10 and TGF-β (96). Since myristic acid promotes the production of EPA and DHA and is negatively correlated with air pollutants, the lower levels of myristic acid in the GDM hair samples could potentially link air pollution exposure to reduced anti-inflammatory capacity in women with GDM.

2-Hydroxybutyric acid (2-HB) was another hair metabolite that exhibited a lower concentration in GDM from pre-conception to the third trimester and was negatively associated with SO_2_. The results from a meta-analysis and cohort studies have found that maternal exposure to SO_2_ increased the risk of GDM (31, 63, 97, 98). Plasma 2-hydroxybutyric acid has been previously reported to be associated with insulin resistance and type 2 diabetes (99, 100). Research has demonstrated that 2-hydroxybutyric acid was produced from 2-ketobutyric acid in response to oxidative stress-induced glutathione synthesis (100, 101). Elevated oxidative stress may increase methionine catabolism by cystathionine β-synthase to produce cysteine for glutathione synthesis, while 2-ketobutyric acid is generated as a by-product (102). Indeed, a lower level of 2-ketobutyric acid (1^st^ and 2^nd^ trimesters), methionine (all four periods), and cysteine (3^rd^ trimester) were also observed in the hair of women diagnosed with GDM. Since glutathione metabolism could be downregulated by hyperglycemia, a lower level of 2-hydroxybutyric acid may be reflecting poor antioxidant capacity in women with GDM.

### Limitations

Lastly, we found that the spatiotemporal LUR model estimate data of PM_2.5_ and NO_2_ related to hair metabolites were more statistically significant than the classical proximal-distance model. LUR models are trained and validated against measured pollutant concentrations using variables generated from a geographic information system (GIS), such as distance to nearest source, road network density, land use, terrain, population density, and meteorological variables to predict the concentrations at residential locations. The specific limitations of the LUR models used in this study are described elsewhere (54). Despite the fact that the LUR model provides a better basis for air exposure estimation, only PM_2.5_ and NO_2_ LUR models are currently available for the CLIMB study. Furthermore, air pollutant exposures usually have a hysteresis effect, distributed lag non-linear model (DLNM) might be more appropriate to consider the lag time when estimating the effects of air pollutants on metabolites. Future studies should establish LUR models to estimate other air pollutants (PM_10_, SO_2_, CO, and O_3_) and consider delayed effects of air pollution for our Chongqing cohort. We should also validate our findings in a large multicentre cohort with diverse environmental exposures. In addition, it would be beneficial for future studies to consider pollutant exposure beyond the residential setting which was used in this study, expanding to include exposures from participants’ workplaces and outdoor activities. A potential limitation of this study was that lifestyle behaviours such as nutritional intake, physical activity, and stress during pregnancy were not accounted for and may have been a source of residual confounding. Future studies should also validate the significant metabolites by targeted metabolomics using calibration curves to determine their limits of detection (LODs).

## Conclusions

**Figure 7:**
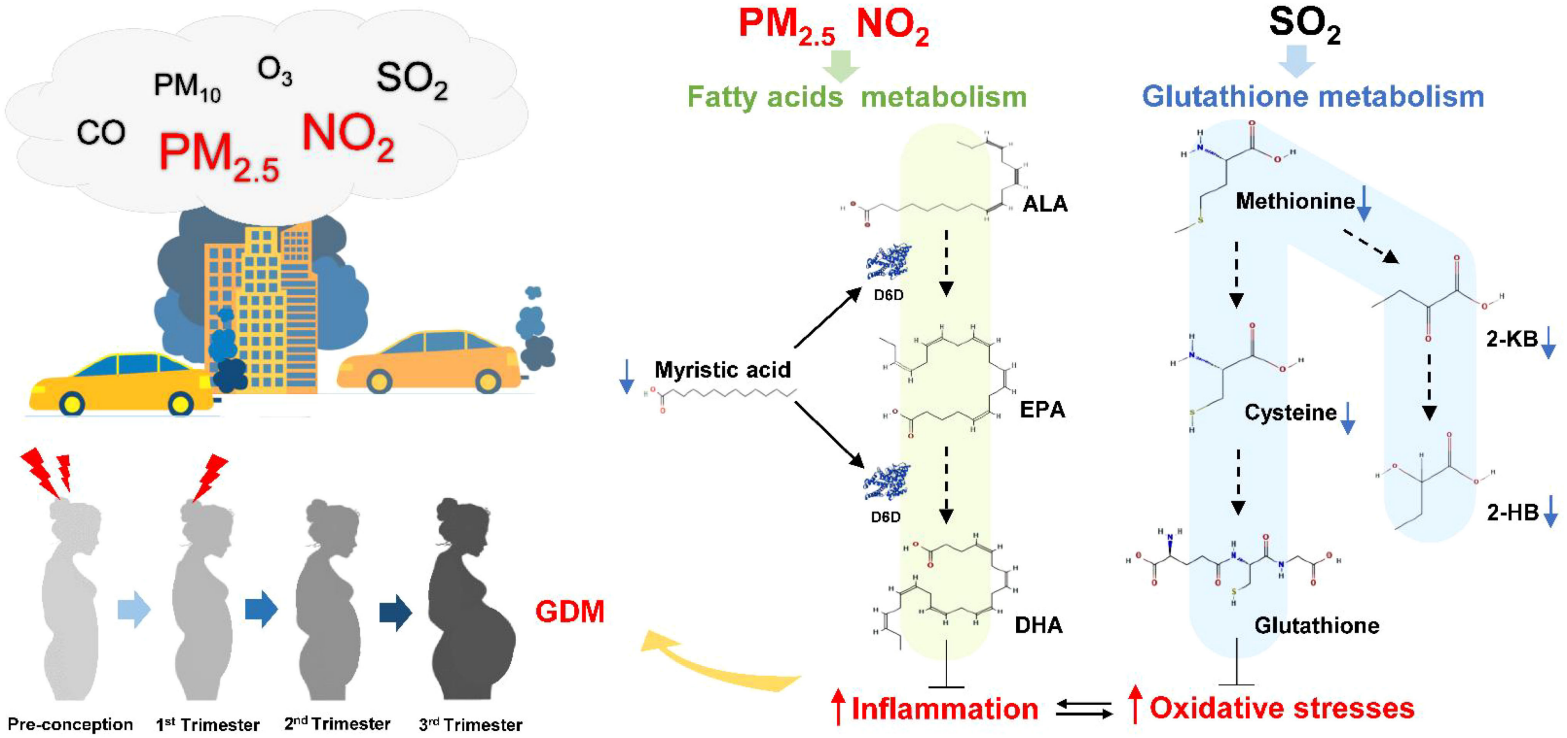
The potential pathways that air pollution exposure influences GDM development through fatty acids and glutathione metabolism. Higher PM_2.5_ and NO_2_ air pollution exposure from pre-conception to early pregnancy are likely to increase pregnant women’s susceptibility to GDM through induced oxidative stress and inflammation. Myristic acid could promote the production of EPA and DHA to compete against inflammation. Methionine is catablised into cysteine and 2-ketobutyric acid (2-KB), which in turn is converted into glutathione and 2-hydroxybutyric acid (2-HB) respectively. The lower level of myristic acid and 2-HB in hair could be related to poor antioxidant and anti-inflammatory capacity in women with GDM. Therefore, increased exposure to air pollutants might promote GDM pathogenesis by downregulating fatty acids and glutathione metabolism. Abbreviations as follows: ALA, α-linolenic acid; EPA, eicosapentaenoic acid; DHA, docosahexaenoic acid; D6D, delta-6 desaturase; 2-HB, 2-hydroxybutyric acid; 2-KB, 2-ketobutyric acid.

This study was the first to investigate the association between environmental pollution, the hair metabolome, and GDM status longitudinally from pre-conception through to the third trimester. We demonstrated that there were significant alterations to the maternal hair metabolome, which were related to later GDM development. Three of these altered metabolites were also associated with maternal air pollution exposure reflecting that exposure to environmental pollutants can increase the risk of GDM. Based on these findings we propose that air pollution contributes to altered metabolism, and potentially an increase in oxidative stress and inflammatory reaction, all of which may contribute to the aetiology of GDM (**Figure 7**). Our findings that the hair metabolome is altered in response to maternal and environmental perturbations suggest that maternal hair could be used to estimate exposure risk factors and better understand the underlying GDM pathophysiology.

## Data availability statement

The raw data supporting the conclusions of this article will be made available by the authors, without undue reservation.

## Ethics statement

The studies involving human participants were reviewed and approved by the Ethics committee of the Chongqing Medical University. The patients/participants provided their written informed consent to participate in this study.

## Author contributions

HQ, PB, JG, HZ, and T-LH conceived the idea and designed the study. YX and TZ take responsibility for the data and sample collection. XZ and YY performed sample preparation and GC-MS analysis. XC, XZ, MJ, AH, and T-LH analyzed data. XC, JS, and T-LH contributed to the writing of the manuscript. RS, PB, HQ, RC, and HZ critically revised the manuscript. All authors contributed to the article and approved the submitted version.

## Funding

This work was supported by the National Natural Science Foundation of China (No. 81971406, 81871185), The 111 Project (Yuwaizhuan (2016)32), Chongqing Science & Technology Commission (cstc2021jcyj-msxmX0213), Chongqing Municipal Education Commission (KJZD-K202100407), Chongqing Health Commission and Chongqing Science & Technology Commission (2021MSXM121, 2020MSXM101).

## Acknowledgments

The authors thank all collaborators for data collection. We also thank all the study participants. The authors are also grateful to support provided by The Chongqing Key Laboratory of Translational Medicine in Major Metabolic Diseases.

## Conflict of interest

The authors declare that the research was conducted in the absence of any commercial or financial relationships that could be construed as a potential conflict of interest.

The handling editor RZ declared a shared affiliation with the authors XC, XZ, YY, YX, TZ, HQ, HZ, T-LH at the time of review.

## Publisher’s note

All claims expressed in this article are solely those of the authors and do not necessarily represent those of their affiliated organizations, or those of the publisher, the editors and the reviewers. Any product that may be evaluated in this article, or claim that may be made by its manufacturer, is not guaranteed or endorsed by the publisher.

## Abbreviations

ALA, α-linolenic acid; BMI, body mass index; CO, carbon monoxide; CLIMB, Complex Lipids in Mothers and Babies; D6D, delta-6 desaturase; DHA, docosahexaenoic acid; EPA, eicosapentaenoic acid; GC-MS, gas chromatography-mass spectrometry; GDM, Gestational diabetes mellitus; LUR, land use regression; MCF, Methyl chloroformate; NO_2_, nitrogen dioxide; O_3_, ozone; PM_2.5_, particulate matter less than 2.5μm in diameter; PM_10_, particulate matter less than 10μm in diameter; SO_2_, sulfur dioxide; UMAP, Uniform Manifold Approximation and Projection; 2-HB, 2-hydroxybutyric acid; 2-KB, 2-ketobutyric acid.
