# Supplementary material for: The relationship between hair metabolites, air pollution exposure and gestational diabetes mellitus: A longitudinal study from pre-conception to third trimester
Source: Front Endocrinol (Lausanne). 2022 Dec 2;13:1060309. doi: 10.3389/fendo.2022.1060309 (PMC9755849; doi:10.3389/fendo.2022.1060309)
Supplement: Supplementary file 1 [file DataSheet_1.docx]

Supplementary Material

**Supplementary Table 1.** List of monitoring sites and monitoring site classification.

| Site ID | Name | Classification |
| --- | --- | --- |
| 1 | Jin-yun-shan | Rural forest site (spatial outlier) |
| 2 | Gao-jia-hua-yuan | Urban traffic site |
| 3 | Tian-sheng | Suburban traffic site |
| 4 | Liang-lu | Urban traffic site |
| 5 | Hu-xi | Urban traffic site |
| 6 | Nan-ping | Urban background site |
| 7 | Tang-jia-tuo | Suburban background site |
| 8 | Cha-yuan | Urban traffic site (Automobile industrial site) |
| 9 | Bai-shi-yi | Urban background site (Airport site) |
| 10 | Jie-fang-bei | Urban centre background site |
| 11 | Yang-jia-ping | Urban background site |
| 12 | Kong-gang | Urban traffic site |
| 13 | Xin-shan-cun | Urban background site |
| 14 | Li-jia | Urban background site |
| 15 | Cai-jia | Urban background site |
| 16 | Yu-xin-jie | Urban background site |
| 17 | Nan-quan | Urban background site |
| 18 | Xie-tai-zi | Urban traffic site |
| 19 | Long-jing-wan | Suburban background site |
| 20 | Long-zhou-wan | Urban traffic site |
| 21 | Shang-qing-si | Urban background site |

**Supplementary Table 2**. Performance of spatiotemporal land use regression models.

|  | COR-R^2^ | MSE-R^2^ |
| --- | --- | --- |
| PM_2.5_ | 0.72 (0.67-0.77) | 0.71 (0.66-0.76) |
| NO_2_ | 0.39 (0.24-0.50) | 0.31 (0.00-0.49) |

COR-R^2^ and MSE-R^2^ are displayed as the mean, with minimum and maximum in parentheses. Abbreviations: COR-R^2^, Pearson’s correlation coefficient, squared; MSE-R^2^, mean-square-error-based-R^2^.

**Supplementary Table 3.** Metabolites between GDM cases and non-GDM controls in unadjusted and adjusted models (age and BMI) in linear mixed model.

| Metabolites | *p-*value | *p-*value  (adjusted) | *q-*value | *q-*value  (adjusted) | Classification |
| --- | --- | --- | --- | --- | --- |
| Cabamic acid | 0.938 | 0.654 | 0.979 | 0.908 | Organic acids |
| 2-Oxobutyric acid | 0.506 | 0.314 | 0.907 | 0.840 | Organic acids |
| Oxalic acid | 0.003 | <0.001 | 0.041 | 0.012 | Organic acids |
| Isobutyl methyl carbote (NIST:72.5%) | 0.510 | 0.353 | 0.907 | 0.840 | Exogenous compounds |
| Imidazole, 4-fluoro- (NIST:71.0%) | 0.687 | 0.230 | 0.953 | 0.840 | Food intake |
| Hexanoic acid (C6_0) | 0.542 | 0.270 | 0.926 | 0.840 | Saturated fatty acids |
| Cyclotetrasiloxane, octamethyl- (NIST:84.4%) | 0.057 | 0.038 | 0.351 | 0.283 | Exogenous compounds |
| 2-Oxovaleric acid | 0.564 | 0.342 | 0.931 | 0.840 | wine intake |
| Nicotimide | 0.675 | 0.545 | 0.953 | 0.889 | Organic acids |
| N-Formyl-d-threo-O-methylthreonine (NIST:67.4%) | 0.001 | 0.049 | 0.027 | 0.321 | Exogenous compounds |
| Pyrazole, 5-methyl-3-(5-nitro-2-furyl)- (NIST:73.7%) | 0.284 | 0.162 | 0.762 | 0.720 | Food additives |
| Azetidine, 1,1'-methylenebis- (NIST:72.1%) | 0.385 | 0.036 | 0.854 | 0.282 | Exogenous compounds |
| 1-tert-Butoxy-2-ethoxyethane (NIST:57.4%) | 0.325 | 0.448 | 0.800 | 0.840 | Organic acids |
| Oxetane, 2,3,4-trimethyl- (NIST: 72.9%) | 0.461 | 0.006 | 0.895 | 0.072 | Exogenous compounds |
| Ethane, 1,1,2,2-tetrachloro- (NIST:97.1%) | 0.921 | 0.911 | 0.979 | 0.970 | Food intake |
| Dimethyl trisulfide (NIST:89.9%) | 0.011 | 0.027 | 0.120 | 0.229 | Food intake |
| Hepta-4,6-diyn-2-ol (NIST:56.7%) | 0.807 | 0.991 | 0.976 | 0.999 | Exogenous compounds |
| Heptane, 2,4-dimethyl- (NIST:84.9%) peak 1 | 0.541 | 0.822 | 0.926 | 0.970 | Food intake |
| Ethane, pentachloro- (NIST:94.1%) | 0.981 | 0.829 | 0.986 | 0.970 | Food intake |
| Malonic acid | 0.144 | 0.001 | 0.554 | 0.020 | Organic acids |
| 1-Pentanone, 1-(4-methylphenyl)- (NIST:87.6%) | 0.843 | 0.899 | 0.976 | 0.970 | Food intake |
| 3-Methyl-2-oxopentanoic acid | 0.403 | 0.464 | 0.862 | 0.840 | wine intake |
| 4-Methyl-2-oxopentanoic acid | 0.947 | 0.698 | 0.979 | 0.909 | Organic acids |
| 2-Propenoic acid, 2-methyl-, (tetrahydro-2-furanyl) methyl ester (NIST:82.7%) | 0.858 | 0.600 | 0.976 | 0.907 | Food intake |
| Ethane, hexachloro-(NIST:96.9%) | 0.745 | 0.995 | 0.957 | 0.999 | Food intake |
| Benzene, 1,3-dichloro- (NIST:94.5%) | 0.744 | 0.938 | 0.957 | 0.992 | Tobacco smoke |
| Dimethyl fumarate (NIST:94.9%) | 0.354 | 0.747 | 0.829 | 0.933 | Medicines |
| Methane, tert-butoxymethoxy- (NIST:75.6%) | 0.275 | 0.079 | 0.762 | 0.486 | gut microbial |
| Methyl 4-oxo-2-pentenoate (NIST:93.7%) | 0.026 | 0.004 | 0.221 | 0.049 | Food additives |
| Levulinic acid | 0.021 | 0.009 | 0.195 | 0.109 | Food additives |
| Cyclopentasiloxane, decamethyl- (NIST:90.4%) | <0.001 | <0.001 | <0.001 | 0.009 | Exogenous compounds |
| 1H,1H,2H,2H-Perfluorooctan-1-ol (NIST:66.1%) | 0.492 | 0.985 | 0.900 | 0.999 | Exogenous compounds |
| Ethyl ether (NIST:68.4%) | 0.001 | <0.001 | 0.026 | 0.009 | Exogenous compounds |
| Glutaric acid, 3-methylbut-2-yl but-2-en-1-yl ester (NIST:70.7%) | 0.148 | 0.333 | 0.554 | 0.840 | Organic acids |
| Fumaric acid | 0.574 | 0.984 | 0.931 | 0.999 | Unsaturated fatty acids |
| Lactic acid | 0.229 | 0.475 | 0.703 | 0.840 | Organic acids |
| Diazene, [1-(2,2-dimethylhydrazino)-2-methylpropyl] ethyl- (NIST:64.4%) | 0.577 | 0.295 | 0.931 | 0.840 | Food intake |
| Propylphosphonic acid, fluoroanhydride, 2-methylpenthyl ester (NIST:57.2%) | 0.160 | 0.140 | 0.572 | 0.688 | Exogenous compounds |
| Glutaric acid | 0.464 | 0.366 | 0.895 | 0.840 | Organic acids |
| Octanoic acid (C8_0) | 0.587 | 0.835 | 0.935 | 0.970 | Saturated fatty acids |
| Dodecane | 0.813 | 0.871 | 0.976 | 0.970 | Organic acids |
| Pyridine, 2-(methylthio)- (NIST:92.6%) | 0.441 | 0.468 | 0.888 | 0.840 | Food intake |
| Benzoic acid | 0.933 | 0.897 | 0.979 | 0.970 | Food additives |
| Itaconic acid | 0.018 | 0.050 | 0.178 | 0.321 | Unsaturated fatty acids |
| Pyrimidine-2,4(1H,3H)-dione, 1-hydroxymethyl-6-methyl- (NIST:73.4%) | 0.053 | 0.082 | 0.351 | 0.491 | Exogenous compounds |
| Citraconic acid | 0.162 | 0.033 | 0.572 | 0.266 | Unsaturated fatty acids |
| 2-Hydroxyisobutyric acid | 0.056 | 0.017 | 0.351 | 0.172 | Organic acids |
| 2-Hydroxybutyric acid | 0.002 | 0.001 | 0.030 | 0.020 | Organic acids |
| 3H-Pyrazol-3-one, 2,4-dihydro-2,5-dimethyl- (NIST:62.8%) | 0.645 | 0.241 | 0.953 | 0.840 | Exogenous compounds |
| Nicotinic acid | 0.547 | 0.454 | 0.926 | 0.840 | Organic acids |
| 3,3-Diaziridinedicarboxylic acid, 1-methyl-, dimethyl ester (NIST:63.0%) | 0.727 | 0.492 | 0.957 | 0.844 | Exogenous compounds |
| 3-Piperidinol, 1-ethyl-6-methyl- (NIST:69.2%) | 0.660 | 0.541 | 0.953 | 0.888 | Food intake |
| Citramalic acid | <0.001 | <0.001 | 0.016 | 0.008 | Organic acids |
| NADP_NADPH | 0.899 | 0.730 | 0.979 | 0.928 | Organic acids |
| 4 Hydroxybenzene sulphonic acid | 0.797 | 0.600 | 0.974 | 0.907 | Exogenous compounds |
| Butanedioyl dihydrazide (NIST:93.4%) peak 2 | 0.874 | 0.623 | 0.976 | 0.907 | Medicines |
| Propioldehyde, diethylhydrazone (NIST:99.0%) | 0.620 | 0.439 | 0.953 | 0.840 | Organic acids |
| 4-(2-Hydroxyethyl)-2,2,6,6-tetramethylpiperidine (NIST:53.2%) | 0.240 | 0.296 | 0.712 | 0.840 | Exogenous compounds |
| Butyl 2-methylbutanoate (NIST:75.3%) | 0.135 | 0.294 | 0.548 | 0.840 | Exogenous compounds |
| Alanine | 0.103 | 0.073 | 0.476 | 0.457 | Amino acids |
| para-Toluic acid | 0.001 | 0.010 | 0.023 | 0.109 | Food additives |
| Benzeneacetic acid, methyl ester (NIST:72.6%) | 0.488 | 0.403 | 0.900 | 0.840 | Organic acids |
| Glyoxylic acid | <0.001 | 0.002 | 0.011 | 0.025 | Organic acids |
| DL-Alanine, N-methyl-N-(byt-3-en-1-yloxycarbonyl)-, hexadecyl ester (NIST:66.7%) | 0.686 | 0.698 | 0.953 | 0.909 | Amino acids and their derivatives |
| Cyclohexasiloxane, dodecamethyl- (NIST:93.2%) | 0.083 | 0.254 | 0.434 | 0.840 | Exogenous compounds |
| Malic acid | 0.744 | 0.928 | 0.957 | 0.986 | TCA cycle and derivatives |
| 1-Aminocyclopentanecarboxylic acid, N-methoxycarbonyl-, hexyl ester (NIST:74.6%) | 0.513 | 0.484 | 0.907 | 0.840 | Exogenous compounds |
| Formic acid, 2-ethylhexyl ester (NIST:82.0%) | 0.227 | 0.370 | 0.703 | 0.840 | Exogenous compounds |
| Glycine | 0.412 | 0.443 | 0.862 | 0.840 | Amino acids |
| 1-Aminocyclopentanecarboxylic acid, N-methoxycarbonyl-, decyl ester (NIST:73.8%) | 0.671 | 0.684 | 0.953 | 0.909 | Exogenous compounds |
| Ethane, diazo- (NIST:75.2%) | 0.042 | 0.044 | 0.321 | 0.306 | Food intake |
| Mecysteine (NIST:65.4%) | 0.720 | 0.968 | 0.957 | 0.999 | Exogenous compounds |
| 1,5-Dimethylbarbituric acid (NIST:76.3%) | 0.646 | 0.601 | 0.953 | 0.907 | Exogenous compounds |
| N-(-Hydroxyethyl)-4-(-hydroxypropyl) piperidine (NIST:55.7%) | 0.568 | 0.723 | 0.931 | 0.928 | Food intake |
| 1,4-Benzenedicarboxaldehyde (NIST:87.1%) | 0.172 | 0.147 | 0.601 | 0.710 | Exogenous compounds |
| Acetic acid ethenyl ester (NIST:88.3%) | 0.682 | 0.483 | 0.953 | 0.840 | Food additives |
| 2-Aminobutyric acid | 0.698 | 0.647 | 0.953 | 0.907 | Amino acids |
| Adipic acid | 0.024 | 0.112 | 0.210 | 0.583 | Saturated fatty acids |
| Caprinoic acid | 0.064 | 0.305 | 0.382 | 0.840 | Organic acids |
| Phenol, 4-[3-(1-perhydroazocinyl) butyl]- (NIST:58.1%) | 0.291 | 0.406 | 0.762 | 0.840 | Exogenous compounds |
| 1,4-phthalenedione, 2-methyl-3-(3-methyl-2-butenyl) -(NIST:52.7%) | 0.095 | 0.157 | 0.453 | 0.720 | Exogenous compounds |
| 5-Thiazoleethanol, 4-methyl- (NIST:94.9%) | 0.649 | 0.439 | 0.953 | 0.840 | Food intake |
| Ethyl 6-methylpyridine-2-carboxylate (NIST:83.1%) | 0.350 | 0.370 | 0.829 | 0.840 | Food intake |
| 1,2,4,5-Tetrazine, 3,6-dimethyl- (NIST:79.9%) | 0.157 | 0.038 | 0.569 | 0.283 | Exogenous compounds |
| 2-Oxoglutaric acid | 0.971 | 0.364 | 0.985 | 0.840 | Organic acids |
| 3-Acetyl-2-oxo-1,3-oxazolidine (NIST:65.4%) | 0.297 | 0.537 | 0.767 | 0.888 | Exogenous compounds |
| 5-Aminoimidazole-4-carboxylic acid, methyl ester (NIST:62.6%) | <0.001 | <0.001 | 0.008 | 0.009 | Exogenous compounds |
| 1-Aminocyclopropane-1-carboxylic acid | 0.001 | 0.001 | 0.019 | 0.024 | Amino acids and their derivatives |
| 1,3-Dioxolan-2-one (NIST:75.7%) | 0.863 | 0.846 | 0.976 | 0.970 | Exogenous compounds |
| 4,4-Dimethyl-5-methylene [1,3] dioxolan-2-one (NIST:53.7%) | 0.904 | 0.907 | 0.979 | 0.970 | Exogenous compounds |
| 3-Octamine (NIST:91.9%) | 0.825 | 0.643 | 0.976 | 0.907 | Exogenous compounds |
| beta-Alanine | 0.181 | 0.020 | 0.617 | 0.186 | Amino acids |
| 2,3-Butanedione (NIST:84.1%) | 0.086 | 0.085 | 0.440 | 0.500 | Food intake |
| Hydrazine, phenyl- (NIST:87.0%) | 0.734 | 0.219 | 0.957 | 0.840 | Tobacco smoke |
| 2-ketoglutamarate | <0.001 | 0.001 | 0.016 | 0.017 | TCA cycle and derivatives |
| l-Valine, N-methoxycarbonyl-, isohexyl ester2 (NIST:59.4%) | 0.293 | 0.580 | 0.762 | 0.905 | Amino acids and their derivatives |
| Cycloheptasiloxane, tetradecamethyl- (NIST:83.3%) | 0.095 | 0.290 | 0.453 | 0.840 | Exogenous compounds |
| 1,2-Ethanediamine, N, N-bis(1-methylethyl)- (NIST:86.3%) | 0.856 | 0.668 | 0.976 | 0.909 | Exogenous compounds |
| Methyl 2-(2-amino-4-oxo-3H-pyrimidin-5-yl) acetate (NIST:91.9%) | 0.153 | 0.274 | 0.563 | 0.840 | Exogenous compounds |
| l-Isoleucine, N-methoxycarbonyl-, methyl ester (NIST:91.1%) | 0.772 | 0.867 | 0.971 | 0.970 | Amino acids and their derivatives |
| Leucine | 0.097 | 0.126 | 0.453 | 0.630 | Amino acids |
| Isoleucine | 0.342 | 0.312 | 0.829 | 0.840 | Amino acids |
| Ethane, 1,1,2,2-tetraethoxy-2 (NIST:67.6%) | <0.001 | <0.001 | 0.016 | 0.008 | Food intake |
| l-Leucyl-l-leucine, N, N'-dimethyl-N'-(2-methoxyethoxycarbonyl)-, pentyl ester (NIST:61.2%) | 0.229 | 0.331 | 0.703 | 0.840 | Amino acids and their derivatives |
| 1-Chloroundecane (NIST:86.6%) | 0.013 | 0.281 | 0.136 | 0.840 | Exogenous compounds |
| 1,3-Dioxane, 2-methyl- (NIST:91.8%) peak 1 | 0.842 | 0.438 | 0.976 | 0.840 | Exogenous compounds |
| Alanine, N-methyl-N-ethoxycarbonyl-, isobutyl ester (NIST:69.5%) | 0.001 | 0.001 | 0.019 | 0.016 | Amino acids and their derivatives |
| Acetic acid, (2-methoxyethoxy) -(NIST:85.6%) | 0.973 | 0.390 | 0.985 | 0.840 | Organic acids |
| N-(m-Tolyl)-diethanolamine (NIST:61.6%) | 0.237 | 0.350 | 0.712 | 0.840 | Personal care product exposure |
| Dimethyl aminomalonic acid | 0.092 | 0.096 | 0.453 | 0.537 | Amino acids |
| Norleucine | 0.111 | 0.466 | 0.486 | 0.840 | Amino acids |
| DL-3-Aminoisobutyric acid | 0.556 | 0.846 | 0.930 | 0.970 | Amino acids and their derivatives |
| 4-Aminobutyric acid (GABA) | 0.395 | 0.696 | 0.859 | 0.909 | Amino acids |
| Pyrrolidine-2-one-trans-4,5-dicarboxylic acid, dimethyl ester (NIST:56.8%) | 0.115 | 0.197 | 0.496 | 0.825 | Exogenous compounds |
| (4-Pyridylthio) acetic acid (NIST:71.8%) | 0.043 | 0.040 | 0.323 | 0.283 | Exogenous compounds |
| Aziridine, 2-methyl-3-(1-methylethyl)-1-(2-propenyl)-, trans- (NIST:57.2%) | 0.945 | 0.398 | 0.979 | 0.840 | Exogenous compounds |
| Proline | 0.492 | 0.292 | 0.900 | 0.840 | Amino acids |
| Malic acid peak 2 | 0.544 | 0.856 | 0.926 | 0.970 | TCA cycle and derivatives |
| Dodecanoic acid (C12_0) | 0.736 | 0.956 | 0.957 | 0.999 | Saturated fatty acids |
| L-Cysteine, ethyl ester (NIST:56.6%) | 0.791 | 0.990 | 0.971 | 0.999 | Amino acids and their derivatives |
| Methyl cyclopropylcarbamate (NIST:60.4%) | 0.792 | 0.425 | 0.971 | 0.840 | Exogenous compounds |
| cis-Aconitic acid | 0.411 | 0.964 | 0.862 | 0.999 | TCA cycle and derivatives |
| Ethanol, 2-[4-(1,1-dimethylethyl)-2-methylphenoxy]- (NIST:71.6%) | 0.387 | 0.330 | 0.854 | 0.840 | Food intake |
| O-Acetylserine | 0.860 | 0.463 | 0.976 | 0.840 | Amino acids |
| Cyclooctasiloxane, hexadecamethyl- (NIST:82.0%) | 0.065 | 0.612 | 0.382 | 0.907 | Exogenous compounds |
| Dimethyl phthalate (NIST:87.6%) | 0.110 | 0.097 | 0.486 | 0.537 | Exogenous compounds |
| 1,4-Benzenedicarboxylic acid, dimethyl ester (NIST:96.1%) | 0.051 | 0.307 | 0.351 | 0.840 | Food intake |
| l-Isoleucyl-l-isoleucine, N-methoxycarbonyl-, 2,2,3,3,3-pentafluoropropyl ester (NIST:58.2%) | 0.780 | 0.360 | 0.971 | 0.840 | Amino acids and their derivatives |
| Aspartic acid | 0.511 | 0.645 | 0.907 | 0.907 | Amino acids |
| Citric acid | 0.005 | 0.968 | 0.080 | 0.999 | TCA cycle and derivatives |
| 2,4-Di-tert-butylphenol (derivatization artefact) | 0.373 | 0.645 | 0.839 | 0.907 | Organic acids |
| Dimethyl 2,5-thiophenedicarboxylate (NIST:62.3%) | 0.130 | 0.166 | 0.535 | 0.729 | Exogenous compounds |
| Pyroglutamic acid | 0.288 | 0.216 | 0.762 | 0.840 | Amino acids |
| N-(Carboxymethyl)-L-alanine | 0.248 | 0.220 | 0.713 | 0.840 | Amino acids and their derivatives |
| Azelaic acid | 0.048 | 0.002 | 0.339 | 0.025 | Saturated fatty acids |
| DL-gamma-methyl-ketoglutaramate isomer 1 | 0.931 | 0.364 | 0.979 | 0.840 | Exogenous compounds |
| 2-Aminocaprylic acid, N-methoxycarbonyl-, isohexyl ester (NIST:59.0%) | 0.668 | 0.756 | 0.953 | 0.933 | Organic acids |
| Creatinine | 0.729 | 0.460 | 0.957 | 0.840 | Amino acids and their derivatives |
| Pentanethioic acid, S-ethyl ester (NIST:95.1%) | 0.122 | 0.268 | 0.517 | 0.840 | Exogenous compounds |
| Ethyldiethanolamine, O-acetyl (NIST:84.9%) | 0.862 | 0.687 | 0.976 | 0.909 | Exogenous compounds |
| 9-Anthracenecarboxaldehyde, oxime, (Z)- (NIST:56.1%) | 0.210 | 0.639 | 0.673 | 0.907 | Organic acids |
| Methyl bis(ethoxycarbonyl)methylenamine, N-oxide (NIST:50.8%) | 0.390 | 0.424 | 0.854 | 0.840 | Exogenous compounds |
| Glycine, N-(methoxyoxoacetyl)-, methyl ester (NIST:63.6%) | 0.308 | 0.271 | 0.772 | 0.840 | Amino acids and their derivatives |
| 3-Hydroxybenzoic acid | 0.906 | 0.660 | 0.979 | 0.909 | Organic acids |
| Methyl tetradecanoate (NIST:81.5%) | 0.371 | 0.297 | 0.839 | 0.840 | Food intake |
| 1-Ethyl-3, cis-(1,1-dimethylethyl)-4, cis-methoxycyclohexan-1-ol (NIST:74.1%) | 0.024 | 0.001 | 0.210 | 0.016 | Exogenous compounds |
| 1H-Pyrazole-3-carboxylic acid (1,2,2,6,6-pentamethylpiperidin-4-yl) amide (NIST:86.9%) | 0.432 | 0.668 | 0.883 | 0.909 | Exogenous compounds |
| l-Valine, N-methoxycarbonyl-, pentyl ester2 (NIST:65.5%) | 0.895 | 0.633 | 0.979 | 0.907 | Amino acids and their derivatives |
| Myristoleic acid (C14_1n-5c) | 0.291 | 0.401 | 0.762 | 0.840 | Unsaturated fatty acids |
| l-Alanine, N-(2-methoxyethoxycarbonyl)-, nonyl ester (NIST:53.7%) | 0.647 | 0.533 | 0.953 | 0.887 | Amino acids and their derivatives |
| Methyl bis(ethoxycarbonyl)methylenamine, N-oxide (NIST:50.8%) peak 2 | 0.873 | 0.618 | 0.976 | 0.907 | Exogenous compounds |
| Propanedioic acid, (acetylamino)methyl-, diethyl ester (NIST:71.5%) | 0.841 | 0.632 | 0.976 | 0.907 | gut microbial |
| Myristic acid (C14_0) | 0.006 | 0.002 | 0.085 | 0.025 | Saturated fatty acids |
| Cyclonosiloxane, octadecamethyl (NIST:75.5%) | 0.245 | 0.879 | 0.712 | 0.970 | Exogenous compounds |
| 1H-Pyrrolo[1,2-c] imidazole-1,3(2H)-dione, tetrahydro- (NIST:67.8%) | 0.856 | 0.369 | 0.976 | 0.840 | Food intake |
| Propamide, N-(2,6-dimethylphenyl)-3-(4-morpholyl)- (NIST:69.3%) | 0.048 | 0.040 | 0.339 | 0.283 | Food intake |
| 1H-Tetrazole-1,5-diamine (NIST:88.0%) peak 1 | 0.304 | 0.692 | 0.770 | 0.909 | Exogenous compounds |
| 1,3-Dioxol-2-one (NIST:84.8%) | 0.325 | 0.321 | 0.800 | 0.840 | Exogenous compounds |
| Glutamic acid | 0.672 | 0.592 | 0.953 | 0.907 | gut microbial |
| Methionine | 0.001 | <0.001 | 0.019 | 0.009 | Amino acids |
| Dipicolinic acid | 0.006 | 0.337 | 0.085 | 0.840 | Food intake |
| 4-Amino-4,5(1H)-dihydro-1,2,4-triazole-5-one (NIST:93.4%) | 0.945 | 0.228 | 0.979 | 0.840 | Exogenous compounds |
| 1H-Tetrazole-1,5-diamine (NIST:88.0%) peak 2 | 0.867 | 0.254 | 0.976 | 0.840 | Exogenous compounds |
| Glutathione | 0.416 | 0.643 | 0.864 | 0.907 | Organic acids |
| 1-Methylpyrazol-4-amine (NIST:55.9%) peak 1 | 0.652 | 0.467 | 0.953 | 0.840 | Exogenous compounds |
| 4-Hydroxyphenylacetic acid | 0.057 | 0.309 | 0.351 | 0.840 | Exogenous compounds |
| Glycine, N-(2-methoxy-2-oxoethyl)-, methyl ester (NIST:81.4%) | 0.940 | 0.982 | 0.979 | 0.999 | Amino acids and their derivatives |
| 10-Pentadecenoic acid (C15_1n-5c) | 0.147 | 0.254 | 0.554 | 0.840 | Unsaturated fatty acids |
| 2-Diethylamino-2-methylpropane-1,3-diol, diethyl ether (NIST:61.6%) | 0.974 | 0.971 | 0.985 | 0.999 | Food intake |
| L-Glutamic acid, dimethyl ester (NIST:55.7%) | 0.975 | 1.000 | 0.985 | 1.000 | Amino acids and their derivatives |
| N-Acetylglutamic acid | 0.402 | 0.558 | 0.862 | 0.895 | Amino acids |
| Pentadecanoic acid (C15_0) | 0.001 | 0.016 | 0.026 | 0.169 | Saturated fatty acids |
| 1,4-Oxathiin, 2,3-dihydro-6-methyl- (NIST:68.5%) | 0.721 | 0.460 | 0.957 | 0.840 | Exogenous compounds |
| Pyridine, 3-fluoro- (NIST:75.2%) | 0.077 | 0.018 | 0.422 | 0.172 | Food intake |
| Phenylalanine | 0.488 | 0.676 | 0.900 | 0.909 | Amino acids |
| cis-4-Hydroxyproline | 0.052 | 0.031 | 0.351 | 0.263 | Amino acids |
| Cysteine | 0.011 | 0.483 | 0.120 | 0.840 | Amino acids |
| Diethanolamine, 3TMS derivative (NIST:71.4%) | 0.254 | 0.354 | 0.723 | 0.840 | Personal care product exposure |
| 1,3-Dioxolane-4,5-dione (NIST:77.2%) | 0.942 | 0.907 | 0.979 | 0.970 | Exogenous compounds |
| Palmitic acid (C16_0) | 0.001 | 0.018 | 0.027 | 0.172 | Saturated fatty acids |
| D-Norleucine, N-ethoxycarbonyl-, isohexyl ester (NIST:57.6%) | 0.868 | 0.842 | 0.976 | 0.970 | Amino acids and their derivatives |
| 5-Isopropyl-2,4-imidazolidinedione (NIST:82.3%) peak 2 | 0.545 | 0.909 | 0.926 | 0.970 | Exogenous compounds |
| 1-Methylpyrazol-4-amine (NIST:55.9%) peak 2 | 0.041 | 0.571 | 0.320 | 0.896 | Organic acids |
| Methyl anthranilate (NIST:57.2%) | 0.083 | 0.239 | 0.434 | 0.840 | Food intake |
| Palmitelaidic acid (C16_1n-9c) | 0.137 | 0.473 | 0.548 | 0.840 | Unsaturated fatty acids |
| 10,13-dimethyltetradecanoic acid (C17_0) | 0.006 | <0.001 | 0.084 | 0.014 | Saturated fatty acids |
| 3-Hydroxyisoxazole-5-(N-isopropyl) carboxamide (NIST:55.8%) | 0.791 | 0.697 | 0.971 | 0.909 | Exogenous compounds |
| 1,4-piperazinediacetic acid, 2-oxo-, dimethyl ester (NIST:69.7%) | 0.943 | 0.472 | 0.979 | 0.840 | Exogenous compounds |
| Metamphetamine, N-Pentafluoropropionyl- (NIST:58.3%) | 0.147 | 0.117 | 0.554 | 0.599 | Medicines |
| 4,6(1H)-Pyrimidinedione, 3,4,5,6-tetrahydro-2-imino- (NIST:96.5%) | 0.108 | 0.213 | 0.486 | 0.840 | Exogenous compounds |
| Dammar-22-en-3-ol, 20,24-epoxy-24-methyl-, acetate, (3,24S)- (NIST:72.6%) | 0.903 | 0.645 | 0.979 | 0.907 | Exogenous compounds |
| l-Valine, N-methoxycarbonyl-, hexyl ester (NIST:74.4%) | 0.715 | 0.815 | 0.957 | 0.970 | Amino acids and their derivatives |
| 1,2-Propanediol, 1-acetate (NIST:60.9%) | 0.029 | 0.008 | 0.237 | 0.099 | Exogenous compounds |
| Phthalic acid, cyclobutyl isobutyl ester (NIST:78.8%) | 0.265 | 0.266 | 0.746 | 0.840 | Food intake |
| 10-Heptadecenoic acid (C17_1n-7t) | 0.613 | 0.184 | 0.953 | 0.793 | Unsaturated fatty acids |
| Ethamine, 2-methoxy-N-(2-methoxyethyl)-N-methyl- (NIST:77.9%) | 0.597 | 0.483 | 0.941 | 0.840 | Exogenous compounds |
| 1,3-Benzenediol, O, O'-di(trans-3-trifluoromethylcinmoyl)- (NIST:82.9%) | 0.277 | 0.389 | 0.762 | 0.840 | Food additives |
| 1-Butene-3-one, dimethylhydrazone (NIST:61.3%) | 0.823 | 0.394 | 0.976 | 0.840 | Exogenous compounds |
| 9-Heptadecenoic acid (C17_1n-8t) | 0.078 | 0.339 | 0.422 | 0.840 | Unsaturated fatty acids |
| 3-[3-(4-Hydroxyphenyl)-3-oxoprop-1-en-1-yl]-6-methylchromen-4-one (NIST:62.5%) | 0.889 | 0.882 | 0.979 | 0.970 | Exogenous compounds |
| Benzenepropanoic acid, 3,5-bis(1,1-dimethylethyl)-4-hydroxy-, methyl ester (NIST:81.8%) | 0.011 | 0.024 | 0.120 | 0.213 | Organic acids |
| n-Propyl acetate (NIST:99.8%) | 0.143 | 0.001 | 0.554 | 0.020 | Food additives |
| Margaric acid (C17_0) | 0.007 | 0.001 | 0.091 | 0.016 | Saturated fatty acids |
| [1,2,3] Triazolo[4,5-e] [1,4] diazepine-5,8-dione, 1-benzyl-1,4,6,7-tetrahydro- (NIST:72.4%) | 0.175 | 0.155 | 0.603 | 0.720 | Exogenous compounds |
| l-Norleucine, N-methoxycarbonyl-, butyl ester (NIST:77.7%) | 0.788 | 0.767 | 0.971 | 0.933 | Amino acids and their derivatives |
| l-Leucyl-l-alanine, N-methoxycarbonyl-, methyl ester (NIST:81.5%) | 0.694 | 0.604 | 0.953 | 0.907 | Amino acids and their derivatives |
| 1-Methylpyridin(2H)-2-one-5-carboxylic acid, methyl ester (NIST:62.0%) | 0.242 | 0.331 | 0.712 | 0.840 | Exogenous compounds |
| di-(-phenylisopropyl) amine (NIST:74.0%) | 0.343 | 0.238 | 0.829 | 0.840 | Exogenous compounds |
| 1H-Tetrazaborole, 4,5-dihydro-1,4,5-trimethyl- (NIST:70.8%) | 0.712 | 0.412 | 0.957 | 0.840 | Exogenous compounds |
| l-Norvalyl-l-norvaline, N-methoxycarbonyl-, heptyl ester (NIST: 61.7%) | 0.437 | 0.508 | 0.888 | 0.853 | Amino acids and their derivatives |
| 2-Aminoethanol, N, O-diacetyl- (NIST:83.4%) | 0.775 | 0.473 | 0.971 | 0.840 | Exogenous compounds |
| L-Valine, methyl ester (NIST:57.4%) | 0.481 | 0.635 | 0.900 | 0.907 | Amino acids and their derivatives |
| DBP | 0.477 | 0.556 | 0.900 | 0.895 | Organic acids |
| l-Proline, N-methoxycarbonyl-, hexyl ester (NIST:88.3%) | 0.589 | 0.505 | 0.935 | 0.853 | Amino acids and their derivatives |
| N,3-Diethyl-3-heptamine (NIST:64.3%) | 0.623 | 0.446 | 0.953 | 0.840 | Exogenous compounds |
| Diethylboryl--heptanolacta(N-B) m (NIST:67.6%) | 0.945 | 0.510 | 0.979 | 0.853 | Exogenous compounds |
| Linoleic acid (C18_2n-6,9c) | 0.612 | 0.095 | 0.953 | 0.537 | Unsaturated fatty acids |
| 2-Phenyl-4-iodo-1,2,3-triazole (NIST:60.5%) | 0.445 | 0.451 | 0.891 | 0.840 | Exogenous compounds |
| l-Isoleucylglycine, N-methoxycarbonyl-, methyl ester (NIST:71.8%) | 0.771 | 0.762 | 0.971 | 0.933 | Amino acids and their derivatives |
| l-Alanyl-l-proline, N-methoxycarbonyl-, methyl ester (NIST:68.3%) | 0.551 | 0.762 | 0.927 | 0.933 | Amino acids and their derivatives |
| Hydrazine, 1,1-dimethyl-2-pentyl- (NIST:82.3%) | 0.349 | 0.332 | 0.829 | 0.840 | Tobacco smoke |
| N-(5-Methoxymethyl-7-oxo-3,7-dihydro- [1,2,4] triazolo[1,5-a] pyrimidin-2-yl)-acetamide (NIST:61.2%) | 0.071 | 0.302 | 0.402 | 0.840 | Food intake |
| d-Proline, N-methoxycarbonyl-, undecyl ester (NIST:65.9%) | 0.710 | 0.890 | 0.957 | 0.970 | Amino acids and their derivatives |
| Methyl octyl phthalate (NIST:78.1%) | 0.243 | 0.323 | 0.712 | 0.840 | Exogenous compounds |
| Diethyltrisulphide (NIST:72.3%) | 0.369 | 0.149 | 0.839 | 0.710 | Food intake |
| Glycyl-l-leucine, N-methoxycarbonyl-, methyl ester (NIST:84.9%) | 0.453 | 0.890 | 0.893 | 0.970 | Amino acids and their derivatives |
| 2-Pentenoic acid, 4-oxo-, methyl ester, (Z)- (NIST:63.1%) | 0.183 | 0.730 | 0.617 | 0.928 | Organic acids |
| l-Prolylglycine, N-methoxycarbonyl-, 2,2,2-trifluoroethyl ester (NIST:87.0%) | 0.829 | 0.835 | 0.976 | 0.970 | Amino acids and their derivatives |
| 4-Oxazolidinecarboxylic acid, 2-(1,1-dimethylethyl)-3-formyl-, methyl ester, (2R-cis)- (NIST:63.3%) | 0.467 | 0.680 | 0.895 | 0.909 | Exogenous compounds |
| Ornithine | 0.303 | 0.568 | 0.770 | 0.896 | Amino acids |
| 4-Methyl [1] benzothieno[3,2-b] pyridine (NIST:57.6%) | 0.676 | 0.415 | 0.953 | 0.840 | Exogenous compounds |
| Octadecane, 1-isocyato- (NIST:67.5%) | 0.570 | 0.188 | 0.931 | 0.799 | Food intake |
| Cyclohexaneacetic acid, -methyl--propyl-, methyl ester (NIST:58.1%) | 0.671 | 0.443 | 0.953 | 0.840 | Organic acids |
| l-Norleucyl-l-norleucine, N-methoxycarbonyl-, methyl ester (NIST:76.3%) | 0.362 | 0.565 | 0.839 | 0.896 | Amino acids and their derivatives |
| l-Proline, N-methoxycarbonyl-, isohexyl ester 2 (NIST:74.1%) | 0.474 | 0.555 | 0.900 | 0.895 | Amino acids and their derivatives |
| Pyrrolo[1,2-a] pyrazine-1,4-dione, hexahydro-3-(2-methylpropyl)- (NIST:69.2%) | 0.690 | 0.403 | 0.953 | 0.840 | Exogenous compounds |
| Glycine, N-methyl-N-methoxycarbonyl-, hexyl ester (NIST:82.0%) | 0.522 | 0.437 | 0.912 | 0.840 | Amino acids and their derivatives |
| 3-Pyridinecarboxamide, 1-oxide (NIST:68.3%) | 0.823 | 0.722 | 0.976 | 0.928 | Exogenous compounds |
| 1,3-Cyclopentanedione, 4-hydroxy-2-methyl- (NIST:94.2%) | 0.656 | 0.452 | 0.953 | 0.840 | Exogenous compounds |
| 2,3-Furandione, dihydro-4,4-dimethyl- (NIST:56.5%) | 0.825 | 0.803 | 0.976 | 0.963 | Food intake |
| l-Leucine, N-methoxycarbonyl-, hexyl ester (NIST:59.9%) | 0.590 | 0.568 | 0.935 | 0.896 | Amino acids and their derivatives |
| Glycyl-l-proline, n-butoxycarbonyl-, methyl ester (NIST:55.6%) | 0.374 | 0.898 | 0.839 | 0.970 | Amino acids and their derivatives |
| l-Leucine, N-methoxycarbonyl-, pentyl ester (NIST:62.4%) | 0.451 | 0.874 | 0.893 | 0.970 | Amino acids and their derivatives |
| Glycyl-l-proline, N-methoxycarbonyl-, methyl ester (NIST:84.0%) | 0.217 | 0.628 | 0.689 | 0.907 | Amino acids and their derivatives |
| Lysine | 0.778 | 0.776 | 0.971 | 0.939 | Amino acids |
| Histidine | 0.924 | 0.733 | 0.979 | 0.928 | Amino acids |
| Benzonitrile, 4-[[(2.5-dioxo-1-pyrrolidinyl) oxy] carbonyl]- (NIST:83.7%) | 0.570 | 0.004 | 0.931 | 0.057 | Food intake |
| Arachidic acid (C20_0) | 0.803 | 0.790 | 0.976 | 0.953 | Saturated fatty acids |
| Piperazine, 1,4-dinitroso- (NIST:83.8%) | 0.463 | 0.878 | 0.895 | 0.970 | Medicines |
| Butamide, N-(4-hydroxyphenyl)- (NIST:67.1%) | 0.618 | 0.725 | 0.953 | 0.928 | Medicines |
| 2-n-Hexylphenol (NIST:70.4%) | 0.762 | 0.866 | 0.971 | 0.970 | Exogenous compounds |
| l-Norleucine, N-methoxycarbonyl-, hexyl ester (NIST:72.8%) | 0.519 | 0.269 | 0.912 | 0.840 | Amino acids and their derivatives |
| Carbonic acid, allyl butyl ester (NIST:66.0%) | 0.500 | 0.654 | 0.907 | 0.908 | Exogenous compounds |
| Heneicosanoic acid (C21_0) | 0.995 | 0.502 | 0.995 | 0.853 | Saturated fatty acids |
| d-Prolyl-d-proline, N-methoxycarbonyl-, methyl ester (NIST:87.0%) | 0.910 | 0.951 | 0.979 | 0.998 | Amino acids and their derivatives |
| Hydrazine, N, N-dimethyl-N'-diethylboryl- (NIST:94.4%) | 0.684 | 0.767 | 0.953 | 0.933 | Tobacco smoke |
| d-Proline, N-methoxycarbonyl-, isohexyl ester (NIST:90.4%) | 0.920 | 0.748 | 0.979 | 0.933 | Amino acids and their derivatives |
| 2-Isopropyl-4-methoxy-oxazolidine-3-carboxylic acid, methyl ester (NIST:57.3%) | 0.195 | 0.435 | 0.641 | 0.840 | Exogenous compounds |
| 2-Pyrrolidinone, 3-[bis(trimethylsilyl)amino]-1-(trimethylsilyl)- (NIST:73.8%) | 0.974 | 0.994 | 0.985 | 0.999 | Organic acids |
| l-Valine, N-methoxycarbonyl-, pentyl ester (NIST:73.1%) | 0.628 | 0.818 | 0.953 | 0.970 | Amino acids and their derivatives |
| 6-[2-Hexahydroazepino-1-hydroxyethyl]-8-methyl-2-p-methylphenylquinoline (NIST:94.7%) | 0.354 | 0.943 | 0.829 | 0.994 | Exogenous compounds |
| Benzo[c]cinnoline-2-carboxylic acid, methyl ester (NIST:61.2%) | 0.071 | 0.752 | 0.402 | 0.933 | Organic acids |
| Glutamic acid, N-(2-methylbutyryl)-, dimethyl ester (NIST:55.9%) | 0.658 | 0.619 | 0.953 | 0.907 | gut microbial |
| Azepan-1-yl-acetic acid, methyl ester (NIST:63.2%) | 0.002 | 0.871 | 0.032 | 0.970 | Exogenous compounds |
| L-Phenylalanine, N-acetyl-, methyl ester (NIST:66.1%) | 0.286 | 0.745 | 0.762 | 0.933 | Amino acids and their derivatives |
| l-Leucine, N-methoxycarbonyl-, heptyl ester (NIST:54.0%) | 0.199 | 0.492 | 0.647 | 0.844 | Amino acids and their derivatives |
| 1-Bromo-2-benzyloxybenzene (NIST:68.9%) | 0.739 | 0.466 | 0.957 | 0.840 | Exogenous compounds |
| l-Proline, N-methoxycarbonyl-, octyl ester (NIST:89.1%) | 0.428 | 0.854 | 0.881 | 0.970 | Amino acids and their derivatives |
| 2-Pyrrolidinecarboxylic acid, 1-(2-phenylethyl)-, methyl ester (NIST:56.6%) | 0.089 | 0.111 | 0.446 | 0.583 | Organic acids |
| 4,4,5-Trimethyl-6,8-dioxa-3-thia-bicyclo (3,2,1) ctane (NIST:61.4%) | 0.008 | 0.050 | 0.102 | 0.321 | Exogenous compounds |
| l-Allylglycine, N-ethoxycarbonyl-, ethyl ester (NIST:50.0%) | 0.406 | 0.435 | 0.862 | 0.840 | Amino acids and their derivatives |
| Phthalic acid, di(hept-3-yl) ester (NIST:82.0%) | 0.128 | 0.161 | 0.535 | 0.720 | Food intake |
| Pyrrolo[1,2-a] pyrazine-3-propamide, 2,3,6,7,8,8a-hexahydro-1,4-dioxo- (NIST:57.9%) | 0.982 | 0.384 | 0.986 | 0.840 | Exogenous compounds |
| Allylamine, bis(2-methoxyethyl)- (NIST:61.9%) | 0.023 | 0.688 | 0.210 | 0.909 | Exogenous compounds |
| N,4-Diethyl-4-heptamine (NIST:60.7%) | 0.972 | 0.859 | 0.985 | 0.970 | Exogenous compounds |
| d-Proline, N-methoxycarbonyl-, pentyl ester (NIST:68.5%) | 0.189 | 0.112 | 0.627 | 0.583 | Amino acids and their derivatives |

| Metabolites | *p*-value | *q*-value |
| --- | --- | --- |
| [1,2,3] Triazolo[4,5-e] [1,4] diazepine-5,8-dione, 1-benzyl-1,4,6,7-tetrahydro- (NIST:72.4%) | <0.001 | <0.001 |
| Leucine | <0.001 | <0.001 |
| 2-Phenyl-4-iodo-1,2,3-triazole (NIST:60.5%) | <0.001 | 0.001 |
| l-Proline, N-methoxycarbonyl-, isohexyl ester 2 (NIST:74.1%) | <0.001 | 0.001 |
| 2-Hydroxybutyric acid | <0.001 | 0.001 |
| Phenylalanine | <0.001 | 0.001 |
| Isoleucine | <0.001 | 0.001 |
| l-Prolylglycine, N-methoxycarbonyl-, 2,2,2-trifluoroethyl ester (NIST:87.0%) | <0.001 | 0.001 |
| Ethane, 1,1,2,2-tetraethoxy-2 (NIST:67.6%) | <0.001 | 0.004 |
| Pyrrolo[1,2-a] pyrazine-1,4-dione, hexahydro-3-(2-methylpropyl)- (NIST:69.2%) | <0.001 | 0.007 |
| Aspartic acid | <0.001 | 0.007 |
| Methionine | 0.001 | 0.009 |
| N-(Carboxymethyl)-L-alanine | 0.001 | 0.010 |
| Citramalic acid | 0.001 | 0.014 |
| Ethyl ether (NIST:68.4%) | 0.002 | 0.017 |
| Pyridine, 3-fluoro- (NIST:75.2%) | 0.002 | 0.023 |
| Aziridine, 2-methyl-3-(1-methylethyl)-1-(2-propenyl)-, trans- (NIST:57.2%) | 0.003 | 0.027 |
| Proline | 0.003 | 0.028 |
| 1H-Tetrazole-1,5-diamine (NIST:88.0%) | 0.004 | 0.030 |
| l-Isoleucine, N-methoxycarbonyl-, methyl ester (NIST:91.1%) | 0.004 | 0.035 |
| 2-Aminobutyric acid (From old library) | 0.005 | 0.038 |
| L-Glutamic acid, dimethyl ester (NIST:55.7%) | 0.005 | 0.038 |
| 2-Hydroxyisobutyric acid | 0.006 | 0.038 |
| l-Isoleucylglycine, N-methoxycarbonyl-, methyl ester (NIST:71.8%) | 0.006 | 0.038 |
| Benzeneacetic acid, methyl ester (NIST:72.6%) | 0.007 | 0.041 |

**Supplementary Table 4.** Significant metabolites between GDM cases and non-GDM controls in pre-conception by logistic regression adjusted by confounding factors (age and BMI).

**Supplementary Table 5.** Significant metabolites between GDM cases and non-GDM controls in first trimester by logistic regression adjusted by confounding factors (age and BMI).

| Metabolites | *p*-value | *q*-value |
| --- | --- | --- |
| Leucine | <0.001 | <0.001 |
| [1,2,3] Triazolo [4,5-e] [1,4] diazepine-5,8-dione, 1-benzyl-1,4,6,7-tetrahydro- (NIST:72.4%) | <0.001 | <0.001 |
| 2-Hydroxybutyric acid | <0.001 | <0.001 |
| Citramalic acid | <0.001 | <0.001 |
| Phenylalanine | <0.001 | <0.001 |
| Isoleucine | <0.001 | <0.001 |
| 2-Phenyl-4-iodo-1,2,3-triazole (NIST:60.5%) | <0.001 | <0.001 |
| Aspartic acid | <0.001 | <0.001 |
| 2-Hydroxyisobutyric acid | <0.001 | <0.001 |
| l-Proline, N-methoxycarbonyl-, isohexyl ester 2 (NIST:74.1%) | <0.001 | <0.001 |
| Proline | <0.001 | <0.001 |
| l-Prolylglycine, N-methoxycarbonyl-, 2,2,2-trifluoroethyl ester (NIST:87.0%) | <0.001 | <0.001 |
| Ethane, 1,1,2,2-tetraethoxy-2 (NIST:67.6%) | <0.001 | <0.001 |
| 16.7644 min N-(Carboxymethyl)-L-alanine | <0.001 | <0.001 |
| Ethyl ether (NIST:68.4%) | <0.001 | <0.001 |
| Benzeneacetic acid, methyl ester (NIST:72.6%) | <0.001 | <0.001 |
| Alanine | <0.001 | <0.001 |
| Levulinic acid | <0.001 | <0.001 |
| Pyrrolo[1,2-a] pyrazine-1,4-dione, hexahydro-3-(2-methylpropyl)- (NIST:69.2%) | <0.001 | <0.001 |
| Pyrimidine-2,4(1H,3H)-dione, 1-hydroxymethyl-6-methyl- (NIST:73.4%) | <0.001 | <0.001 |
| Pyroglutamic acid | <0.001 | <0.001 |
| l-Isoleucylglycine, N-methoxycarbonyl-, methyl ester (NIST:71.8%) | <0.001 | <0.001 |
| L-Glutamic acid, dimethyl ester (NIST:55.7%) | <0.001 | <0.001 |
| Methionine | <0.001 | <0.001 |
| Propanedioic acid, (acetylamino)methyl-, diethyl ester (NIST:71.5%) | <0.001 | 0.001 |
| l-Isoleucine, N-methoxycarbonyl-, methyl ester (NIST:91.1%) | <0.001 | 0.001 |
| Glutaric acid | <0.001 | 0.001 |
| Creatinine | <0.001 | 0.001 |
| 10,13-dimethyltetradecanoic acid (C17_0) (From old library) | 0.001 | 0.002 |
| Benzenepropanoic acid, 3,5-bis(1,1-dimethylethyl)-4-hydroxy-, methyl ester (NIST:81.8%) | 0.001 | 0.002 |
| Ethyldiethanolamine, O-acetyl (NIST:84.9%) | 0.001 | 0.002 |
| Oxalic acid | 0.001 | 0.002 |
| Glycine | 0.001 | 0.002 |
| 4,6(1H)-Pyrimidinedione, 3,4,5,6-tetrahydro-2-imino- (NIST:96.5%) | 0.001 | 0.002 |
| 1-Ethyl-3, cis-(1,1-dimethylethyl)-4,cis-methoxycyclohexan-1-ol (NIST:74.1%) | 0.001 | 0.003 |
| 1,2-Ethanediamine, N, N-bis(1-methylethyl)- (NIST:86.3%) | 0.001 | 0.003 |
| Glutaric acid, 3-methylbut-2-yl but-2-en-1-yl ester (NIST:70.7%) | 0.001 | 0.003 |
| 10-Pentadecenoic acid (C15_1n-5c) | 0.001 | 0.003 |
| Citraconic acid | 0.001 | 0.003 |
| di-(-phenylisopropyl) amine (NIST:74.0%) | 0.002 | 0.004 |
| Myristic acid (C14_0) | 0.002 | 0.004 |
| 1,3-Benzenediol, O, O'-di(trans-3-trifluoromethylcinmoyl)- (NIST:82.9%) | 0.004 | 0.008 |
| N-Acetylglutamic acid | 0.004 | 0.009 |
| 2-Aminobutyric acid (From old library) | 0.005 | 0.010 |
| 9-Heptadecenoic acid (C17_1n-8t) | 0.006 | 0.012 |
| N-Formyl-d-threo-O-methylthreonine (NIST:67.4%) | 0.006 | 0.012 |
| Azelaic acid | 0.006 | 0.012 |
| Glyoxylic acid | 0.008 | 0.014 |
| Caprinoic acid | 0.008 | 0.016 |
| Margaric acid (C17_0) | 0.010 | 0.018 |
| 3-Octamine (NIST:91.9%) | 0.010 | 0.018 |
| Acetic acid ethenyl ester (NIST:88.3%) | 0.012 | 0.020 |
| 2-Oxobutyric acid | 0.016 | 0.027 |
| Methane, tert-butoxymethoxy- (NIST:75.6%) | 0.020 | 0.034 |
| para-Toluic acid | 0.020 | 0.034 |
| Pyrrolidine-2-one-trans-4,5-dicarboxylic acid, dimethyl ester (NIST:56.8%) | 0.021 | 0.034 |
| Methyl tetradecanoate (NIST:81.5%) | 0.021 | 0.034 |
| Lysine | 0.021 | 0.034 |
| n-Propyl acetate (NIST:99.8%) | 0.022 | 0.035 |
| 2-Oxovaleric acid | 0.023 | 0.036 |
| Methyl 4-oxo-2-pentenoate (NIST:93.7%) | 0.025 | 0.037 |
| Dimethyl trisulfide (NIST:89.9%) | 0.025 | 0.037 |
| N-(5-Methoxymethyl-7-oxo-3,7-dihydro- [1,2,4] triazolo[1,5-a] pyrimidin-2-yl)-acetamide (NIST:61.2%) | 0.026 | 0.037 |
| Methyl 2-(2-amino-4-oxo-3H-pyrimidin-5-yl) acetate (NIST:91.9%) | 0.029 | 0.040 |
| Benzonitrile, 4- [[(2.5-dioxo-1- pyrrolidinyl) oxy] carbonyl]- (NIST:83.7%) | 0.029 | 0.040 |
| 1H-Tetrazole-1,5-diamine (NIST:88.0%) | 0.029 | 0.040 |
| D-Norleucine, N-ethoxycarbonyl-, isohexyl ester (NIST:57.6%) | 0.030 | 0.041 |
| Diethyltrisulphide (NIST:72.3%) | 0.031 | 0.041 |
| 1,4-phthalenedione, 2-methyl-3-(3-methyl-2-butenyl) -(NIST:52.7%) | 0.034 | 0.046 |
| Myristoleic acid (C14_1n-5c) | 0.037 | 0.048 |

**Supplementary Table 6.** Significant metabolites between GDM cases and non-GDM controls in second trimester by logistic regression adjusted by confounding factors (age and BMI).

| Metabolites | *p*-value | *q*-value |
| --- | --- | --- |
| Leucine | <0.001 | <0.001 |
| [1,2,3] Triazolo[4,5-e] [1,4] diazepine-5,8-dione, 1-benzyl-1,4,6,7-tetrahydro- (NIST:72.4%) | <0.001 | <0.001 |
| Phenylalanine | <0.001 | <0.001 |
| 2-Phenyl-4-iodo-1,2,3-triazole (NIST:60.5%) | <0.001 | <0.001 |
| Proline | <0.001 | <0.001 |
| Isoleucine | <0.001 | <0.001 |
| Aspartic acid | <0.001 | <0.001 |
| Alanine | <0.001 | <0.001 |
| 16.7644 min N-(Carboxymethyl)-L-alanine | <0.001 | <0.001 |
| l-Prolylglycine, N-methoxycarbonyl-, 2,2,2-trifluoroethyl ester (NIST:87.0%) | <0.001 | <0.001 |
| Ethane, 1,1,2,2-tetraethoxy-2 (NIST:67.6%) | <0.001 | <0.001 |
| para-Toluic acid | <0.001 | <0.001 |
| l-Proline, N-methoxycarbonyl-, isohexyl ester 2 (NIST:74.1%) | <0.001 | <0.001 |
| 2-Hydroxybutyric acid | <0.001 | <0.001 |
| 2-Hydroxyisobutyric acid | <0.001 | <0.001 |
| di-(-phenylisopropyl) amine (NIST:74.0%) | <0.001 | <0.001 |
| Glyoxylic acid | <0.001 | <0.001 |
| l-Isoleucylglycine, N-methoxycarbonyl-, methyl ester (NIST:71.8%) | <0.001 | <0.001 |
| L-Glutamic acid, dimethyl ester (NIST:55.7%) | <0.001 | <0.001 |
| Creatinine | <0.001 | <0.001 |
| 10,13-dimethyltetradecanoic acid (C17_0) (From old library) | <0.001 | <0.001 |
| Glutaric acid, 3-methylbut-2-yl but-2-en-1-yl ester (NIST:70.7%) | <0.001 | <0.001 |
| Ethyl ether (NIST:68.4%) | <0.001 | <0.001 |
| Glycine | <0.001 | 0.001 |
| Benzenepropanoic acid, 3,5-bis(1,1-dimethylethyl)-4-hydroxy-, methyl ester (NIST:81.8%) | <0.001 | 0.001 |
| Benzeneacetic acid, methyl ester (NIST:72.6%) | <0.001 | 0.001 |
| l-Isoleucine, N-methoxycarbonyl-, methyl ester (NIST:91.1%) | <0.001 | 0.002 |
| Propanedioic acid, (acetylamino)methyl-, diethyl ester (NIST:71.5%) | <0.001 | 0.002 |
| Citramalic acid | <0.001 | 0.002 |
| 3-Methyl-2-oxopentanoic acid | 0.001 | 0.002 |
| Ethyldiethanolamine, O-acetyl (NIST:84.9%) | 0.001 | 0.002 |
| Pyroglutamic acid | 0.001 | 0.002 |
| Lysine | 0.001 | 0.002 |
| N-Formyl-d-threo-O-methylthreonine (NIST:67.4%) | 0.001 | 0.003 |
| 1,2-Ethanediamine, N, N-bis(1-methylethyl)- (NIST:86.3%) | 0.001 | 0.003 |
| 1-Ethyl-3, cis-(1,1-dimethylethyl)-4, cis-methoxycyclohexan-1-ol (NIST:74.1%) | 0.001 | 0.004 |
| Methyl 4-oxo-2-pentenoate (NIST:93.7%) | 0.001 | 0.004 |
| Metamphetamine, N-Pentafluoropropionyl- (NIST:58.3%) | 0.002 | 0.005 |
| Pyrrolo[1,2-a] pyrazine-1,4-dione, hexahydro-3-(2-methylpropyl)- (NIST:69.2%) | 0.002 | 0.005 |
| Methionine | 0.002 | 0.005 |
| 1,3-Benzenediol, O, O'-di(trans-3-trifluoromethylcinmoyl)- (NIST:82.9%) | 0.002 | 0.006 |
| D-Norleucine, N-ethoxycarbonyl-, isohexyl ester (NIST:57.6%) | 0.002 | 0.006 |
| N-Acetylglutamic acid | 0.002 | 0.006 |
| 2-Aminobutyric acid (From old library) | 0.002 | 0.006 |
| Acetic acid ethenyl ester (NIST:88.3%) | 0.003 | 0.008 |
| Diazene, [1-(2,2-dimethylhydrazino)-2-methylpropyl] ethyl- (NIST:64.4%) | 0.003 | 0.008 |
| 2-Oxovaleric acid | 0.003 | 0.008 |
| Myristic acid (C14_0) | 0.004 | 0.010 |
| 10-Pentadecenoic acid (C15_1n-5c) | 0.005 | 0.011 |
| Dimethyl trisulfide (NIST:89.9%) | 0.006 | 0.015 |
| Azelaic acid | 0.007 | 0.016 |
| Alanine, N-methyl-N-ethoxycarbonyl-, isobutyl ester (NIST:69.5%) | 0.008 | 0.018 |
| Ornithine | 0.008 | 0.018 |
| Dipicolinic acid | 0.009 | 0.020 |
| Levulinic acid | 0.010 | 0.020 |
| 3-Octamine (NIST:91.9%) | 0.010 | 0.021 |
| N-(5-Methoxymethyl-7-oxo-3,7-dihydro- [1,2,4] triazolo[1,5-a] pyrimidin-2-yl)-acetamide (NIST:61.2%) | 0.011 | 0.023 |
| Norleucine | 0.013 | 0.027 |
| 14.9814 min Dimethyl aminomalonic acid | 0.017 | 0.033 |
| 1-tert-Butoxy-2-ethoxyethane (NIST:57.4%) | 0.017 | 0.034 |
| 2-Oxobutyric acid | 0.018 | 0.034 |
| l-Valine, N-methoxycarbonyl-, isohexyl ester2 (NIST:59.4%) | 0.020 | 0.038 |
| Nicotinic acid | 0.021 | 0.039 |
| Cyclotetrasiloxane, octamethyl- (NIST:84.4%) | 0.021 | 0.039 |
| (4-Pyridylthio) acetic acid (NIST:71.8%) | 0.025 | 0.044 |
| 2-Diethylamino-2-methylpropane-1,3-diol, diethyl ether (NIST:61.6%) | 0.025 | 0.044 |
| 9-Heptadecenoic acid (C17_1n-8t) | 0.025 | 0.044 |
| Cyclopentasiloxane, decamethyl- (NIST:90.4%) | 0.027 | 0.046 |
| beta-Alanine | 0.029 | 0.049 |

**Supplementary Table 7.** Significant metabolites between GDM cases and non-GDM controls in third trimester by logistic regression adjusted by confounding factors (age and BMI).

| Metabolites | *p*-value | *q*-value |
| --- | --- | --- |
| [1,2,3] Triazolo[4,5-e] [1,4] diazepine-5,8-dione, 1-benzyl-1,4,6,7-tetrahydro- (NIST:72.4%) | <0.001 | <0.001 |
| Leucine | <0.001 | <0.001 |
| l-Prolylglycine, N-methoxycarbonyl-, 2,2,2-trifluoroethyl ester (NIST:87.0%) | <0.001 | <0.001 |
| Proline | <0.001 | <0.001 |
| Alanine | <0.001 | <0.001 |
| Phenylalanine | <0.001 | <0.001 |
| 2-Phenyl-4-iodo-1,2,3-triazole (NIST:60.5%) | <0.001 | <0.001 |
| Aspartic acid | <0.001 | <0.001 |
| Benzenepropanoic acid, 3,5-bis(1,1-dimethylethyl)-4-hydroxy-, methyl ester (NIST:81.8%) | <0.001 | <0.001 |
| di-(-phenylisopropyl) amine (NIST:74.0%) | <0.001 | <0.001 |
| Glycine | <0.001 | <0.001 |
| Isoleucine | <0.001 | <0.001 |
| l-Proline, N-methoxycarbonyl-, isohexyl ester 2 (NIST:74.1%) | <0.001 | <0.001 |
| Ethane, 1,1,2,2-tetraethoxy-2 (NIST:67.6%) | <0.001 | <0.001 |
| Glutaric acid, 3-methylbut-2-yl but-2-en-1-yl ester (NIST:70.7%) | <0.001 | <0.001 |
| Alanine, N-methyl-N-ethoxycarbonyl-, isobutyl ester (NIST:69.5%) | <0.001 | <0.001 |
| 2-Hydroxyisobutyric acid | <0.001 | <0.001 |
| Methyl 4-oxo-2-pentenoate (NIST:93.7%) | <0.001 | <0.001 |
| 1,2-Ethanediamine, N, N-bis(1-methylethyl)- (NIST:86.3%) | <0.001 | <0.001 |
| N-Formyl-d-threo-O-methylthreonine (NIST:67.4%) | <0.001 | <0.001 |
| 16.7644 min N-(Carboxymethyl)-L-alanine | <0.001 | <0.001 |
| Creatinine | <0.001 | <0.001 |
| L-Glutamic acid, dimethyl ester (NIST:55.7%) | <0.001 | <0.001 |
| Propanedioic acid, (acetylamino)methyl-, diethyl ester (NIST:71.5%) | <0.001 | <0.001 |
| l-Isoleucylglycine, N-methoxycarbonyl-, methyl ester (NIST:71.8%) | <0.001 | <0.001 |
| para-Toluic acid | <0.001 | <0.001 |
| 2-Hydroxybutyric acid | <0.001 | <0.001 |
| Levulinic acid | <0.001 | 0.001 |
| Ethyl ether (NIST:68.4%) | <0.001 | 0.001 |
| 1-Ethyl-3, cis-(1,1-dimethylethyl)-4, cis-methoxycyclohexan-1-ol (NIST:74.1%) | 0.001 | 0.001 |
| 10,13-dimethyltetradecanoic acid (C17_0) (From old library) | 0.001 | 0.001 |
| l-Isoleucine, N-methoxycarbonyl-, methyl ester (NIST:91.1%) | 0.001 | 0.001 |
| Azelaic acid | 0.001 | 0.001 |
| 4,6(1H)-Pyrimidinedione, 3,4,5,6-tetrahydro-2-imino- (NIST:96.5%) | 0.001 | 0.001 |
| Benzeneacetic acid, methyl ester (NIST:72.6%) | 0.001 | 0.002 |
| Metamphetamine, N-Pentafluoropropionyl- (NIST:58.3%) | 0.002 | 0.002 |
| Glutaric acid | 0.002 | 0.002 |
| Lysine | 0.002 | 0.003 |
| Acetic acid ethenyl ester (NIST:88.3%) | 0.003 | 0.003 |
| 2-Aminobutyric acid (From old library) | 0.003 | 0.004 |
| D-Norleucine, N-ethoxycarbonyl-, isohexyl ester (NIST:57.6%) | 0.004 | 0.004 |
| 10-Pentadecenoic acid (C15_1n-5c) | 0.004 | 0.004 |
| 14.9814 min Dimethyl aminomalonic acid | 0.005 | 0.005 |
| Norleucine | 0.007 | 0.007 |
| Glyoxylic acid | 0.007 | 0.007 |
| 1,4-Benzenedicarboxaldehyde (NIST:87.1%) | 0.008 | 0.008 |
| Ethyldiethanolamine, O-acetyl (NIST:84.9%) | 0.009 | 0.008 |
| 9-Heptadecenoic acid (C17_1n-8t) | 0.010 | 0.009 |
| 2-Oxovaleric acid | 0.010 | 0.009 |
| Citramalic acid | 0.013 | 0.011 |
| 4-Methyl-2-oxopentanoic acid | 0.015 | 0.013 |
| Ornithine | 0.017 | 0.014 |
| Diazene, [1-(2,2-dimethylhydrazino)-2-methylpropyl] ethyl- (NIST:64.4%) | 0.017 | 0.014 |
| 3-Methyl-2-oxopentanoic acid | 0.017 | 0.014 |
| N-Acetylglutamic acid | 0.017 | 0.014 |
| 1,3-Dioxol-2-one (NIST:84.8%) | 0.018 | 0.014 |
| Myristic acid (C14_0) | 0.018 | 0.014 |
| 3-Octamine (NIST:91.9%) | 0.018 | 0.014 |
| 1H-Tetrazole-1,5-diamine (NIST:88.0%) peak 1 | 0.019 | 0.014 |
| Dipicolinic acid | 0.019 | 0.014 |
| l-Valine, N-methoxycarbonyl-, isohexyl ester2 (NIST:59.4%) | 0.021 | 0.015 |
| Benzonitrile, 4-[[(2.5-dioxo-1-pyrrolidinyl) oxy] carbonyl]- (NIST:83.7%) | 0.023 | 0.016 |
| Cysteine | 0.025 | 0.018 |
| Pyroglutamic acid | 0.025 | 0.018 |
| Dimethyl trisulfide (NIST:89.9%) | 0.032 | 0.022 |
| Pyrrolo[1,2-a] pyrazine-1,4-dione, hexahydro-3-(2-methylpropyl)- (NIST:69.2%) | 0.035 | 0.024 |
| l-Norleucyl-l-norleucine, N-methoxycarbonyl-, methyl ester (NIST:76.3%) | 0.036 | 0.024 |
| Methyl anthranilate (NIST:57.2%) | 0.036 | 0.024 |
| 1-tert-Butoxy-2-ethoxyethane (NIST:57.4%) | 0.037 | 0.024 |
| Methionine | 0.038 | 0.024 |
| N-(5-Methoxymethyl-7-oxo-3,7-dihydro- [1,2,4] triazolo[1,5-a]pyrimidin-2-yl)-acetamide (NIST:61.2%) | 0.039 | 0.024 |
| d-Proline, N-methoxycarbonyl-, pentyl ester (NIST:68.5%) | 0.040 | 0.025 |
| 1-Methylpyridin(2H)-2-one-5-carboxylic acid, methyl ester (NIST:62.0%) | 0.042 | 0.025 |
| Margaric acid (C17_0) | 0.044 | 0.026 |
| Hydrazine, 1,1-dimethyl-2-pentyl- (NIST:82.3%) | 0.047 | 0.027 |
| l-Allylglycine, N-ethoxycarbonyl-, ethyl ester (NIST:50.0%) | 0.047 | 0.027 |
| l-Norvalyl-l-norvaline, N-methoxycarbonyl-, heptyl ester (NIST: 61.7%) | 0.048 | 0.027 |
| L-Valine, methyl ester (NIST:57.4%) | 0.048 | 0.027 |
| 2-ketoglutamarate | 0.048 | 0.027 |
| Methyl tetradecanoate (NIST:81.5%) | 0.049 | 0.027 |
